# Cooperative Hedgehog/GLI and JAK/STAT signaling drives immunosuppressive tryptophan/kynurenine metabolism via synergistic induction of IDO1 in skin cancer

**DOI:** 10.1186/s12964-025-02101-6

**Published:** 2025-02-17

**Authors:** Dominik P. Elmer, Georg Stockmaier, Sandra Grund-Gröschke, Victoria Strobl, Hieu-Hoa Dang, Markus Wiederstein, David Licha, Anna Strobl, Anna Eglseer, Christina Sternberg, Suzana Tesanovic, Wolfgang Gruber, Florian Wolff, Richard Moriggl, Angela Risch, Roland Reischl, Christian G. Huber, Peter W. Krenn, Nikolaus Fortelny, Jutta Horejs-Hoeck, Fritz Aberger

**Affiliations:** 1 Department of Biosciences and Medical Biology, Cancer Cluster Salzburg, Paris Lodron University of Salzburg, Hellbrunner Strasse 34, Salzburg, 5020 Austria; 2 Center for Tumor Biology and Immunology, Paris Lodron University of Salzburg, Salzburg, Austria; 3 Department of Pathology, Medical University of Vienna, Vienna, Austria; 4 Unit of Laboratory Animal Pathology, University of Veterinary Medicine Vienna, Vienna, Austria; 5 Institute of Biochemistry, University of Kiel, Kiel, Germany; 6 Institute of Animal Breeding and Genetics, University of Veterinary Medicine Vienna, Vienna, Austria

**Keywords:** Hedgehog signaling, Skin cancer, GLI transcription factors, Immune evasion, Indoleamine 2,3-dioxygenase 1, Interleukin 6, Interferon gamma, Signal Transducer and activator of transcription (STAT) proteins

## Abstract

**Background:**

Pharmacological targeting of Hedgehog (HH)/GLI has proven effective for certain blood, brain and skin cancers including basal cell carcinoma (BCC). However, limited response rates and the development of drug resistance call for improved anti-HH therapies that take synergistic crosstalk mechanisms and immune evasion strategies into account. In previous work, we demonstrated that cooperation of HH/GLI and Interleukin 6 (IL6)/STAT3 signaling drives BCC growth. Whether synergistic HH-IL6 signaling promotes BCC via the activation of immune evasion mechanisms remained unclear.

**Methods:**

HH-IL6 regulated immunosuppressive genes such as indoleamine 2,3-dioxygenase 1 (IDO1) were identified by gene expression profiling. IDO1 expression was evaluated in human BCC and melanoma models by qPCR and Western blot analyses. The *cis*-regulatory region of *IDO1* was interrogated for HH-IL6-regulated GLI and STAT transcription factor binding and epigenetic modifications by targeted chromatin-immunoprecipitation and bisulfite pyrosequencing. Functional analyses of the immunosuppressive effects of IDO1 involved HPLC-MS measurements of its metabolites and the assessment of T cell proliferation via flow cytometry. Bioinformatic analyses of GLI-STAT cooperation were conducted on published bulk and single-cell RNA-seq data of human BCC and melanoma patients.

**Results:**

We identified IDO1 as a target gene of cooperative GLI-STAT activity in BCC and melanoma. GLI1 and STAT3 transcription factors synergistically enhanced IDO1 expression by jointly binding to the *cis*-regulatory region of *IDO1* and by increasing active chromatin marks at the histone level. In human melanoma cells, inhibition of GLI1 expression prevented the induction of IDO1 expression in response to IL6/STAT3 and IFNγ/STAT1 signaling. Pharmacological targeting of HH/GLI signaling reduced IDO1 expression, resulting in decreased production of the immunosuppressive metabolite kynurenine. Further, inhibition of GLI1 enhanced the efficacy of the selective IDO1 inhibitor epacadostat and rescued T cell proliferation by attenuating IDO1/kynurenine-mediated immunosuppression. Elevated expression of IDO1 correlated with active HH/GLI and JAK/STAT signaling in skin cancer patients supporting the clinical relevance of the mechanistic data presented.

**Conclusions:**

These results identify the immunosuppressive IDO1-kynurenine pathway as a novel pro-tumorigenic target of oncogenic GLI and STAT1/STAT3 cooperation. Our data suggest simultaneous pharmacological targeting of these signaling axes as rational combination therapy in melanoma and non-melanoma skin cancers.

**Supplementary Information:**

The online version contains supplementary material available at 10.1186/s12964-025-02101-6.

## Background

The Hedgehog/glioma-associated oncogene homolog (HH/GLI) signaling pathway plays a causal role in several human malignancies such as skin, brain and blood cancers. Its regulation involves the release of repressive mechanisms, compartmentalization in the primary cilium, proteolytic processing and numerous post-translational modifications (for detailed reviews see [1–3]) as well as crosstalk with other oncogenic pathways modulating its oncogenic activity [4]. Canonical HH/GLI signaling is initiated by the binding of secreted HH protein to its receptor Patched (PTCH), allowing Smoothened (SMO) to translocate to the primary cilium. There, active SMO initiates a signaling cascade that results in high levels of active GLI transcription factors that drive the expression of HH target genes, which, when aberrantly activated, are associated with cancer development(for reviews see [1–3]). Notably, SMO-independent activation of GLI transcription factors can also drive oncogenic transformation (reviewed in [5]).

Genetic activation of HH/GLI signaling in epidermal cells causes basal cell carcinoma (BCC), a very frequent non-melanoma skin cancer with 3–4 million new cases per year in the US [6]. In BCC, genetic loss of PTCH1 function or gain of function mutations in SMO results in ligand-independent constitutive HH/GLI signaling and skin carcinogenesis (reviewed in [7]). Several small molecule inhibitors targeting SMO (SMOi) were approved for treating locally advanced BCC and acute myeloid leukemia (reviewed in [8, 9]). Despite the therapeutic efficacy of SMOi, drug resistance, insufficient response rates and severe adverse effects call for improved strategies such as combinations with immunotherapeutic approaches [10–12]. Recently, the FDA approved the anti-PD1 blocking antibody cemiplimab for severe BCC [13, 14]. This breakthrough is consistent with growing evidence that HH/GLI signaling drives immune evasion mechanisms [15–17].

In melanoma, HH/GLI regulation involves interactions with the RAS-RAF-MEK-ERK signaling axis, thereby promoting SMO-independent GLI1 activity. In addition, PI3K-AKT-mTOR signaling can induce GLI activation, enhancing melanoma progression. Furthermore, the SOX-BRD4 transcriptional complex induces the expression of GLI1 and GLI2 in human melanoma cells (reviewed in [16, 18]).

Aside from RAF inhibitors [19, 20], immune checkpoint blockers (ICB) have been approved for the treatment of advanced melanoma with impressive success for responding patients [21, 22]. However, response rates to anti-PD1 blocking antibodies typically do not exceed 40%, indicating the existence of immune evasive and resistance mechanisms [23].

Resistance to immune checkpoint blockers can involve tryptophan catabolism and the production of immunosuppressive kynurenine metabolites abrogating T cell responses [24]. Elevated expression of indoleamine 2,3-dioxygenase 1 (IDO1) in cancer cells causes local tryptophan starvation and kynurenine production, inhibiting effector T cells and natural killer cells (reviewed in [25–27] and [28, 29]). High kynurenine levels also induce and recruit immunosuppressive regulatory T cells (Tregs) and myeloid-derived suppressor cells (MDSCs) [28, 30, 31]. The potent immunosuppressive role of IDO1 has led to the development of IDO1 inhibitors, currently tested in clinical trials mainly as ICB adjuvant or in triple combination with radiotherapy, though with variable outcome [32–38].

Expression of IDO1 is controlled by inflammatory cytokine pathways such as Interferon gamma (IFNγ) and Interleukin 6 (IL6) [26, 30, 37, 39, 40]. We have recently shown that integration of HH/GLI and IL6/STAT3 signaling drives the expression of HH-IL6 target genes and BCC growth [41]. Building on these data, we here report on the identification of IDO1 as a novel direct GLI-STAT target gene regulated by combined HH/GLI and JAK/STAT signaling. Our studies support a model where simultaneous GLI-STAT activation increases IDO1 levels in skin cancer cells, inhibiting human effector T cells by tryptophan degradation and enhanced kynurenine concentrations. In silico analyses of RNA-seq data sets support this mechanism in melanoma and non-melanoma skin cancer patients. This study describes a novel immunosuppressive mechanism driven by HH/GLI and JAK/STAT signaling via IDO1 activation, with potential implications for combination therapies using IDO1 inhibitors with HH-JAK/STAT and immune checkpoint blockers.

## Methods

### Cell culture

Cells were maintained at 37 °C in an incubator with humidified atmosphere containing 5% CO_2_. Compounds and cytokines are listed in Additional file 1 table S1. HEK293FT cells for transfection experiments were purchased from Invitrogen (RRID: CVCL_6911) and cultured in Dulbecco’s Modified Eagle’s Medium supplemented with 10% fetal bovine serum, 1% penicillin/streptomycin solution, 1% L-glutamine (Merck, Darmstadt, Germany) and 1% non-essential amino acids solution (Thermo Fisher Scientific, Waltham, MA, United States).

Human keratinocyte (HaCaT) cell lines [42] with doxycycline (dox)-inducible GLI1 expression with and without Myc-tagged GLI1 were maintained and induced as described previously [43]. To induce GLI1 expression, HaCaT cells were pre-treated with dox [50 ng/mL] for 24 h and then treated with panJAK inhibitor 1 (JAKi) [1 µM] for another 24 h with IL6 [75 ng/mL] added for the last 22 h.

Human melanoma cell lines WM35 and WM793B were purchased from ATCC and cultured in Dulbecco’s Modified Eagle’s Medium supplemented with 10% fetal bovine serum and 1% penicillin/streptomycin solution (Merck, Darmstadt, Germany). For chemical inhibition of the HH pathway cells were treated for 6 h with either vismodegib [0.5 µM] or HPI-1 [5 µM, 10 µM]. STAT activation and IDO1 expression were induced by addition of 75 ng/mL IL6 or 10 ng/mL IFNγ for 18 h. For generation of conditioned melanoma media for immune cell inhibition experiments, tryptophan (Merck, Darmstadt, Germany) was added to the culture media to a final concentration of 200 µM to allow efficient kynurenine production in response to IDO1 expression.

All studies involving human immune cells were performed in agreement with the guidelines of the World Medical Association’s Declaration of Helsinki. Human peripheral blood mononuclear cells (PBMCs) were isolated from buffy coats of healthy, anonymous donors (provided by the Blood Bank Salzburg, Austria) and cultured as described previously [44]. Proliferation of CD4^+^ and CD8^+^ T cells was induced by stimulating cells with plate bound anti-CD3 [0.1 µg/mL] (Thermo Fisher Scientific, MA, USA) and soluble anti-CD28 [1 µg/mL] (BD Biosciences, NJ, USA).

### Bead-array expression profiling

mRNA expression profiling has been described previously in ref [41]. Expression values were normalized to control and log2 transformed. The heatmap was generated using GraphPad Prism software (version 8.0.2, San Diego, CA, USA).

### RNA-seq data analysis

Bioinformatic analysis of the Bonilla [21] and Atwood [10] RNA-seq data sets was performed in R (version 4.0.4) using the packages data.table (version 1.14.0), ggplot2 (version 3.3.3), pheatmap (version 1.0.12), edgeR (version 3.32.1), limma (version 3.46.0), and umap (version 0.2.7.0). Raw count data from Bonilla et al., 2016 [21] were obtained by contacting the authors. Counts of all genes were then normalized to log2(cpm) values using the functions *calcNormFactors* from edgeR and *voom* from limma. HH and IL6 activity signatures (see Additional file 1 supplementary information) were calculated by first normalizing the expression of each gene using the function *scale* in base R and then calculating the average across genes from each gene set. HH high and low samples were assigned using a cutoff of -0.5. IL6 high and low samples were assigned using a cutoff of 0. A linear model was fitted using the *lm* function in base R, with IDO1 expression encoded as the dependent variable that is explained by two main effects (one for HH activity and one for IL6 activity) plus an interaction effect of both pathways as independent variables.

Correlation analysis in melanoma and non-melanoma skin cancer was done via TNMplot [45] that contains data from healthy, cancer and metastatic tissue. Data was derived from bulk gene chip data and included tumor samples from GEO, GTex, TCGA and TARGET databases.

Single cell data for analyses of GLI and STAT activity in tumor cells was accessed via Single Cell Portal (https://singlecell.broadinstitute.org/single_cell) and included two separate sample sets with one melanoma patient each (for refs. see Additional file 1 supplementary information). Cell type separation was based on original clustering. Tumor cells were grouped for IDO1^pos^ and IDO1^neg^ expressers based on detectable transcript levels. Cells with any detectable IDO1 expression were considered positive. GLI and STAT1 activity was assessed by looking at the expression of GLI1, GLI2 or GLI3 (for GLI activity) and STAT1 and IRF1 (for STAT1 activity), respectively. Cells positive for GLI1, GLI2 or GLI3 were equivalent to having active GLI signaling; cells positive for STAT1 and IRF1 to having active STAT1 signaling.

### Analysis of mRNA and protein expression

Total RNA was isolated following a standard phenol-chloroform extraction protocol using TRI-reagent (Merck, Darmstadt, Germany) with subsequent LiCl (Carl Roth, Karlsruhe, Germany) precipitation. cDNA was synthesized using M-MLV reverse transcriptase (Promega, Mannheim, Germany). Reverse transcription qPCR (RT-qPCR) runs were conducted as described previously [41]. The synergy score was calculated as described by [46].

Standard protocols were used for SDS-PAGE and Western blot analysis of proteins. Relative quantification of Western blot bands was conducted via densitometric image analysis using Image Lab 4.0 software (Bio-Rad, Vienna, Austria). Band intensities of the loading control or total protein were used for normalization.

Primer sequences and antibodies are listed in Additional file 1 supplementary table S2 and S3.

### Promoter analysis and chromatin immunoprecipitation (ChIP)

As referenced in [41] and the Additional file 1 supplementary information, putative GLI binding sites were predicted by the D-Light client-server software package using the matrix of consensus and non-consensus GLI binding site motives and information on STAT binding sites was retrieved from the ENCyclopedia Of DNA Elements (ENCODE) project.

ChIP was performed with the magnetic bead SimpleChIP Kit (Cell Signaling Technology, Boston, MA, USA) according to the manufacturer’s instructions (4 × 10^7 cells per chromatin sample) followed by qPCR as described previously [41].

Pyrosequencing is described in Additional file 1 supplementary information with primer sequences and antibodies listed in supplementary table S2 and S3.

### RNA interference and lentiviral transduction

Production of lentiviral particles by HEK293FT cells using metafectene pro (Biontex Laboratories, Munich, Germany) and transduction of melanoma cells was performed following a published protocol (for reference, modifications and short hairpin RNA (shRNA) constructs see Additional file 1 supplementary information).

### Metabolite extraction and HPLC‑MS analysis

For detailed method parameters see Additional file 1 supplementary information. Briefly, metabolites were extracted from conditioned media by dilution (1:10) in ice-cold methanol (Merck, Darmstadt, Germany) containing 3-nitro-L-tyrosine [5 µM] (Merck, Darmstadt, Germany) as internal standard (ISTD) followed by centrifugation (10 min, 4 °C, 13000 x g). Methanol extracts were diluted 1:5 in Milli-Q water (Merck, Darmstadt, Germany) and subjected to reversed-phase HPLC-MS using an Accela 1250 HPLC system equipped with a Hypersil Gold aQ column and coupled to a QExactive™ quadrupole-Orbitrap™ mass spectrometer (all from Thermo Fisher Scientific, MA, USA). Analyte separation was performed applying a multistep H_2_O - ACN gradient, metabolites were detected operating the QExactive™ mass spectrometer in parallel reaction monitoring. Data were analyzed using the Thermo Xcalibur software (version 3.0.63, Thermo Fisher Scientific, MA, USA). Peak areas of tryptophan and kynurenine were normalized to the ISTD and used for relative quantification.

### Flow cytometry

PBMCs were stained with proliferation dye eFluor 450 [2 µM] (Thermo Fisher Scientific, MA, USA), seeded into anti-CD3 [0.1 µg/mL] (Thermo Fisher Scientific) pre-coated 48-well plates (2.5 × 10^5 cells per well) and 50% conditioned melanoma or control medium were added (total volume 500 µL per well). PBMCs were then treated with anti-CD28 [1 µg/mL] (BD Biosciences, NJ, USA) and incubated for 72 h. After that, PBMCs were stained with the viability dye eFluor 780, anti-CD3-PE, anti-CD4-FITC and anti-CD8-BV510 (manufacturers and dilutions are listed in Additional file 1 supplementary table S3), fixed in 4% paraformaldehyde (Merck, Darmstadt, Germany) and subjected to flow cytometric analysis on a BD FACS Canto II (BD Biosciences, NJ, USA). Flow cytometry data were analyzed with the FlowJo software (BD Biosciences, NJ, USA). The gating strategy is described in Additional file 2 supplementary Fig. S1.

### Statistical analysis

All data sets were tested for normal distribution. Statistically significant differences between two groups were tested using paired student’s t-tests, except for the analysis of human RNA-seq patient samples, where unpaired Welch’s t tests were used, with a confidence level of 95% for all analyses. For multiple comparisons of normally distributed data ANOVA was used. Detailed information on the statistical tests used for each experiment is given in the figure legends. Levels of significance were subdivided into following categories: *p < 0.05; **p < 0.01; ***p < 0.001. Graphs and statistics were generated using GraphPad Prism software (version 8.0.2, San Diego, CA, USA). For each set of replicates the mean ± standard deviation indicated as error bars were depicted in the graphs.

## Results

### Synergistic HH/GLI and IL6/STAT3 signaling induces IDO1 expression in human keratinocytes

**Fig. 1:**
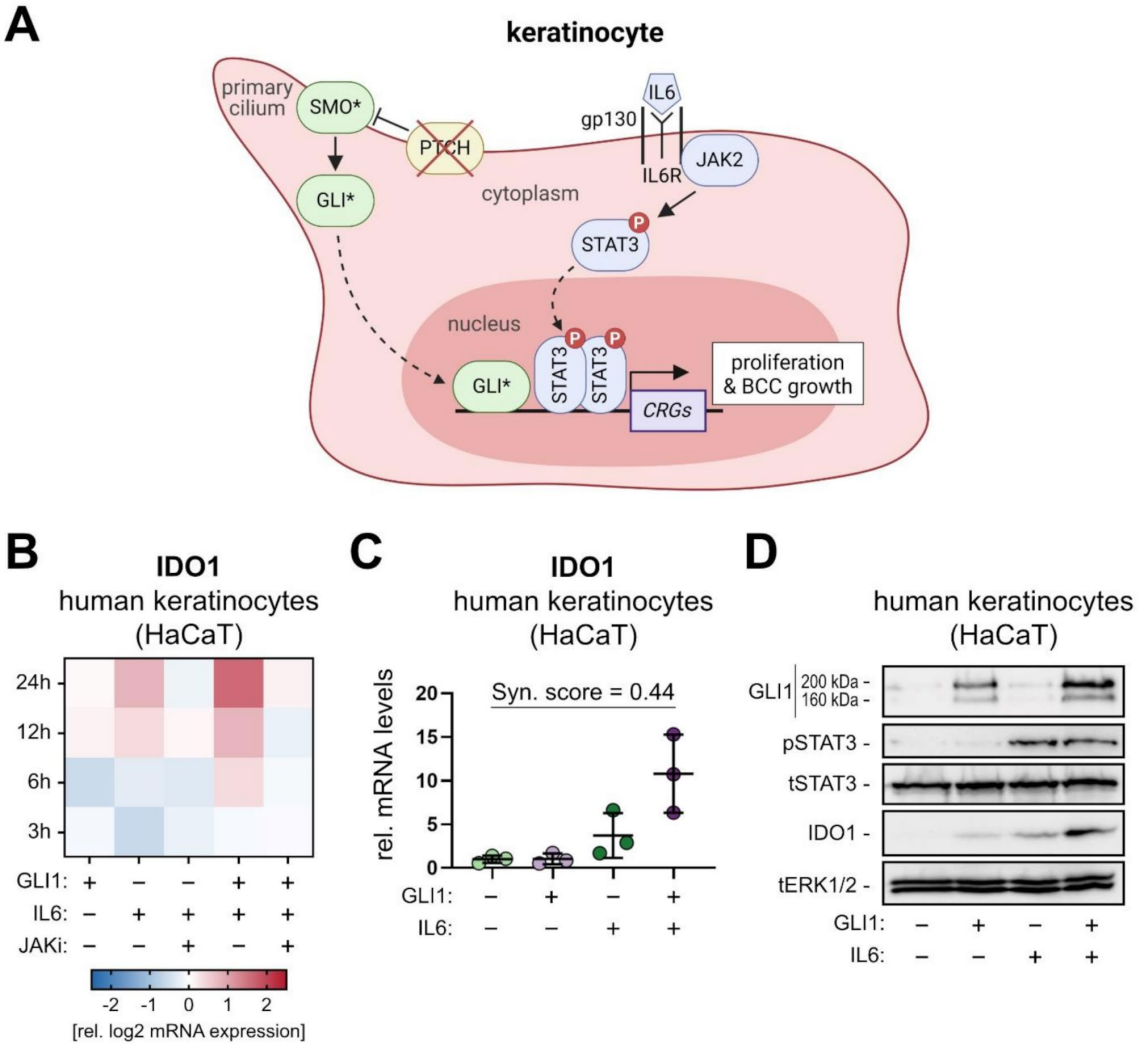
HH/GLI and IL6/STAT3 signaling cooperatively induce the expression of immunosuppressive IDO1. (**A**) Schematic overview of the synergistic interaction of HH/GLI and IL6/STAT3 signaling in keratinocytes driving proliferation and BCC growth. Loss of PTCH allows activation of SMO, which leads to the formation of active GLI transcription factors that translocate into the nucleus (asterisks indicate the active forms of the proteins). In addition, IL6 signaling is initiated by binding of IL6 to its receptor, thereby, STAT3 proteins are phosphorylated via JAK2 and translocate into the nucleus as dimers. Nuclear GLI and STAT3 induce the expression of cooperation response genes (CRGs). (**B**) Heatmap of log2 mRNA expression values of IDO1 measured by bead array expression profiling after 3, 6, 12 and 24 h in dox-inducible GLI1 human HaCaT keratinocytes in the presence or absence of IL6 [75 ng/mL] and panJAK inhibitor 1 (JAKi) [1 µM]. (**C**) IDO1 mRNA expression levels in HaCaT keratinocytes relative to solvent control as measured by qPCR. HaCaT cells were treated for 48 h with dox to induce GLI1 expression, IL6 [75 ng/mL] or a combination of both for the last 18 h (*n* = 3). A synergy score of < 1.0 reflects a more than additive induction of IDO1 expression in response to combined dox-GLI1/IL6-STAT3 activation [46]. (**D**) Representative Western blot analysis of IDO1 expression in HaCaT keratinocytes treated with dox and IL6 as described in (**C**). Active STAT3 signaling was assessed by measuring phospho-STAT3 (pSTAT3). Total ERK1/2 (tERK1/2) protein expression was used as loading control. Two protein bands for GLI1 are visible representing tagged and untagged GLI1 protein. (CRG: cooperation response gene; dox: doxycycline; JAKi: panJAK inhibitor 1; Syn. Score: synergy score; p: phospho; t: total)

In previous work by our group cooperative HH/GLI and IL6/STAT3 signaling have been shown to synergistically activate common cooperation response genes and to drive BCC growth (Fig. 1A) [41]. In light of accumulating evidence that HH/GLI signaling regulates the induction of immunosuppressive mechanisms [16, 17], we interrogated our HH/GLI and proinflammatory IL6/STAT3 mRNA profiling data [41] for novel synergistically regulated HH-IL6 target genes with a known function in immunosuppression and cancer immune evasion. We identified IDO1, a well-documented enzyme involved in cancer immune evasion, as a potentially synergistically induced HH-IL6 target gene in human HaCaT keratinocytes (Fig. 1B). Addition of the JAK inhibitor panJAK inhibitor 1 efficiently suppressed the activation of IDO1 expression, suggesting JAK-dependent synergy of IL6 with HH/GLI signaling (Fig. 1B). We confirmed the gene expression profiling data by qPCR (Fig. 1C, S2A) and Western blotting (Fig. 1D, S2B). To induce GLI1 expression conditionally, cells were pre-treated with dox for 24 h [50 ng/mL] and then treated with IL6 [75 ng/mL] for another 24 h to co-activate JAK/STAT signaling. mRNA levels of IDO1 showed a synergistic upregulation upon activation of combined HH and IL6 signaling with a synergy score of 0.44, indicative of a more than additive activation of target gene expression (Fig. 1C) [46]. Consistently, IDO1 protein levels were also strongly induced upon HH/GLI and IL6/STAT3 pathway activation (Fig. 1D).

### Epigenetic activation of the *cis*-regulatory region of IDO1 upon cooperation of HH/GLI and IL6/STAT3 signaling

**Fig. 2:**
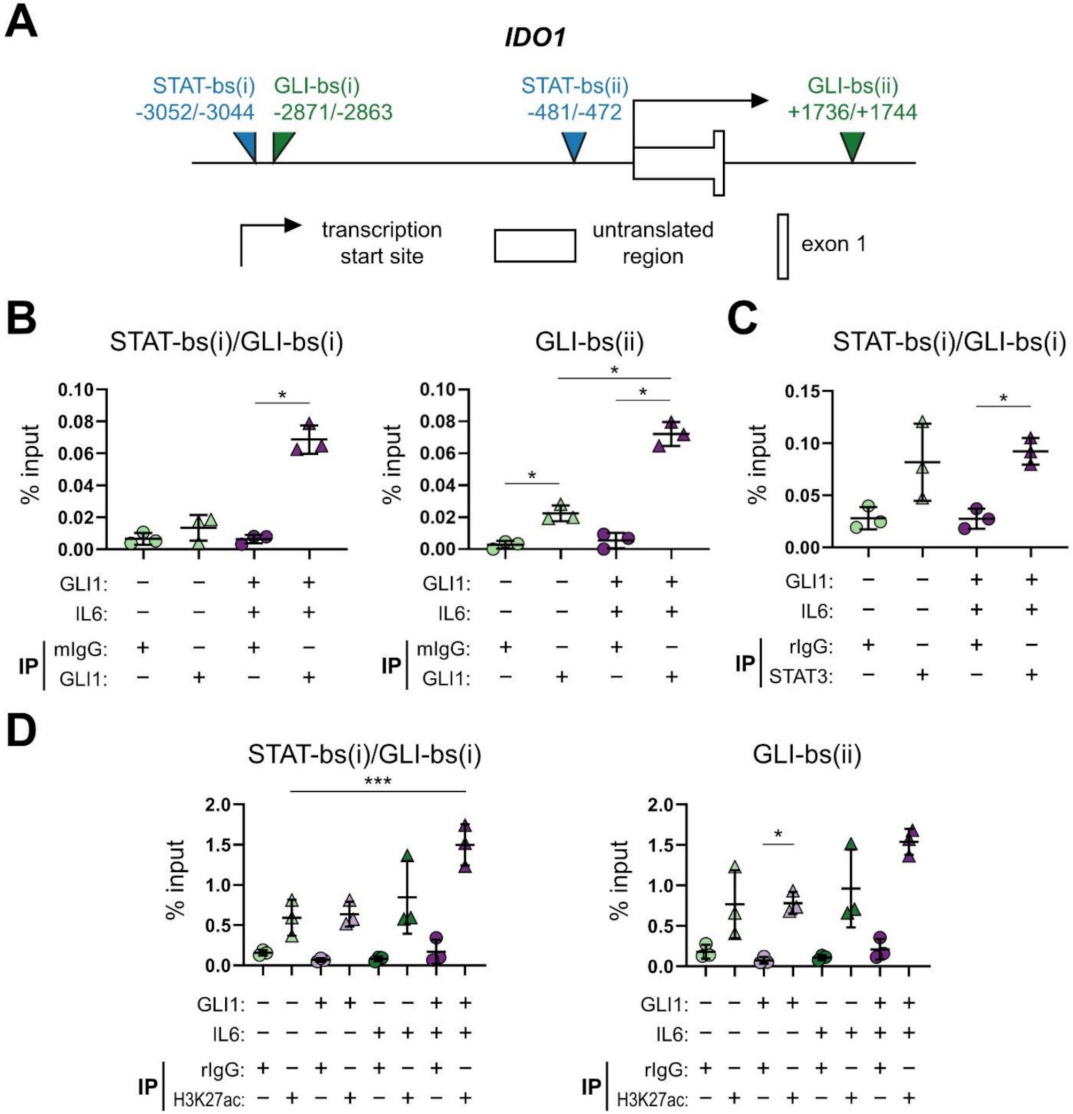
Co-occupancy of the *IDO1cis*-regulatory region by GLI1 and STAT3 promotes an active chromatin state. (**A**) Illustration of the *IDO1cis*-regulatory region with in silico predicted GLI (green) and STAT (blue) binding sites with their position relative to the transcriptional start site (not drawn to scale). (**B-C**) Targeted ChIP analysis of GLI1 and STAT3 showing transcription factor binding to the following predicted sites in the *IDO1cis*-regulatory region: (**B**) GLI1 binding to STAT-bs(i)/GLI-bs(i) as well as GLI-bs(ii). (**C**) STAT3 binding to STAT-bs(i) immediately adjacent to GLI-bs(i) as evidenced by enrichment of the respective binding sequences upon GLI1 or STAT3 ChIP quantified by qPCR as percentage of input chromatin. (**B**, **C**) Human HaCaT keratinocytes expressing myc-tagged GLI1 under dox control were treated for 48 h with dox and 30 min with IL6 [75 ng/mL] to simultaneously activate STAT3 (*n* = 3). (**D**) ChIP analysis of active chromatin as determined by the level of H3K27 histone acetylation on STAT-bs(i)/GLI-bs(i) and GLI-bs(ii) expressed as percentage of input (*n* = 3). Cells were treated as described in (**B**, **C**). One-way ANOVA with Tukey’s multiple-comparison test was used for statistical analysis (*p < 0.05; ***p < 0.001). (GLI-bs: GLI binding site; STAT-bs: STAT binding site, mIgG: mouse IgG; rIgG: rabbit IgG)

Having shown that combined HH/GLI and IL6/STAT3 signaling cooperatively regulate IDO1 expression, we aimed to investigate the molecular basis of signal integration and the transcriptional activation of *IDO1*. We hypothesized that HH-IL6 signal integration converges at the *cis*-regulatory region of *IDO1*, a mechanism we have previously reported for other HH-IL6 cooperation response genes [41]. We first screened for putative GLI binding sites within this region using the in silico binding site prediction tool D‑light. This resulted in the prediction of two putative GLI-binding sites (bs) (GLI-bs(i) and GLI-bs(ii)) at nucleotide position ‑2871 upstream of the transcription start site (TSS) and + 1736 downstream of the TSS, respectively (Fig. 2A). Using human myc-tagged GLI1-inducible HaCaT keratinocytes, we show by ChIP-qPCR that GLI1 binds to both sites upon GLI1 expression or combined activation of HH/GLI and IL6 signaling (Fig. 2B, S3A). Furthermore, we retrieved data on two known STAT-bs in the *IDO1 cis*-regulatory region from the ENCODE database. As shown by a D-light analysis, both STAT binding sites are predicted as sequences recognized and bound by STAT3 as well as STAT1. Notably, STAT-bs(i) is near GLI-bs(i) (within 200 bp; Fig. 2A). Using again a ChIP-qPCR approach, we found STAT3 binding to STAT-bs(i) next to GLI-bs(i) in IL6-treated GLI1 expressing cells (Fig. 2C). Thus, we propose that combined binding and co-occupancy of the *IDO1 cis*-regulatory region by GLI and STAT transcription factors accounts for synergistic activation of IDO1 expression in response to HH-IL6 signaling. Of note, we observed baseline induction of STAT3 in HaCaT cells which could not be increased upon treatment (Fig. 2C).

Additionally, we investigated whether the activity of HH/GLI alone or in combination with IL6/STAT3 signaling affects the epigenetic signature and landscape of the *IDO1 cis*-regulatory elements. To this end, we performed ChIP-qPCR experiments using antibodies specific for H3K27 acetylation, a marker for open active chromatin [47]. Combined activation of both signaling pathways resulted in a significant increase in H3K27 acetylation compared to the control at the STAT-bs(i)/GLI-bs(i) binding region (Fig. 2D), with a similar, yet statistically not significant trend at GLI-bs(ii) (Fig. 2D).

By contrast, combined HH/GLI-IL6 treatment did not alter the CpG methylation status in the region containing STAT-bs(i)/GLI-bs(i) (Fig. S3B, C).

### IL6/STAT3 as well as IFNγ/STAT1-mediated induction of IDO1 requires GLI1 in melanoma cells

**Fig. 3:**
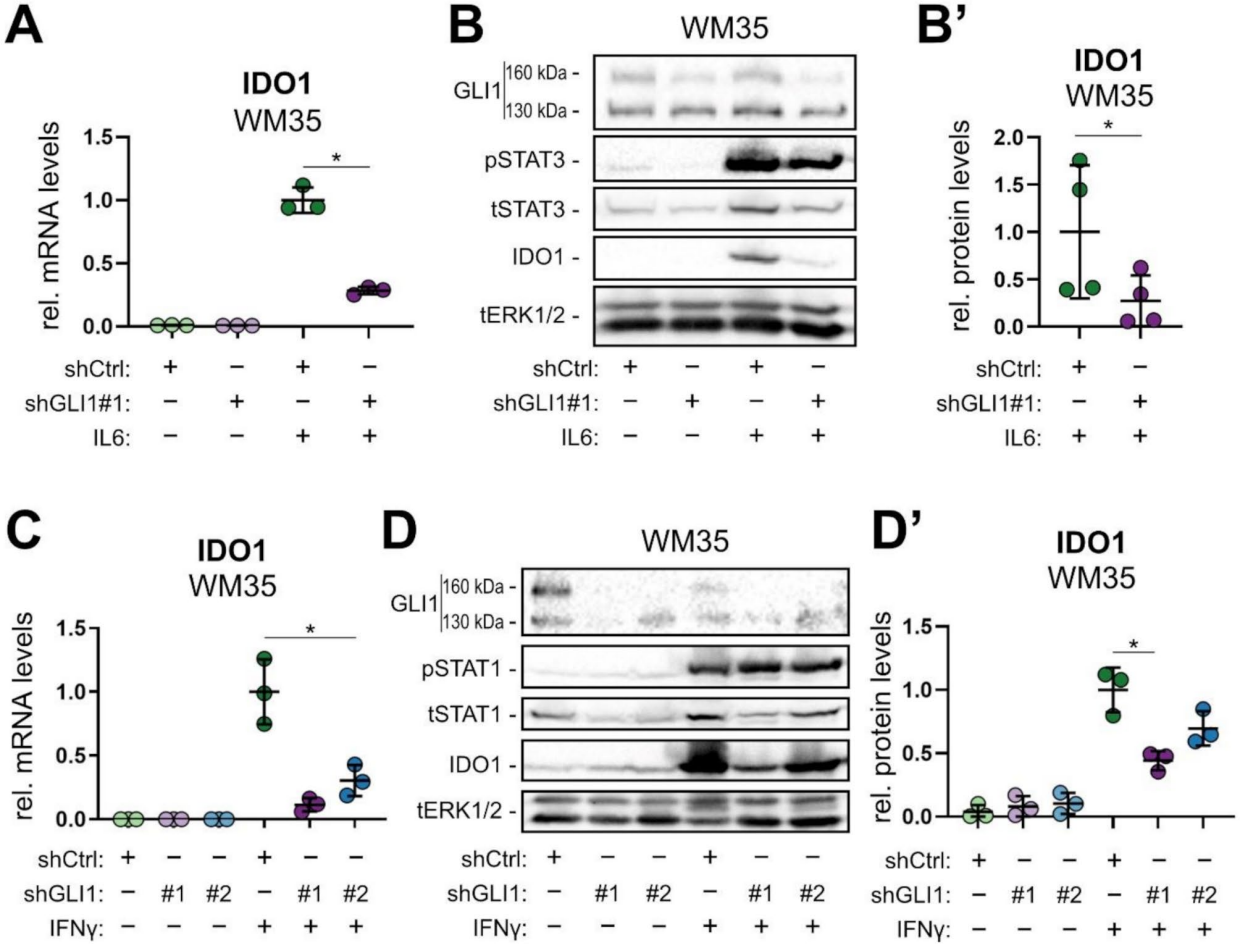
IL6- and IFNγ-induced expression of IDO1 depends on GLI1 function in WM35 human melanoma cells. (**A**) qPCR analysis of IDO1 mRNA levels in WM35 melanoma cells treated with or without IL6 [75 ng/mL], and lentivirally transduced with shGLI1 (shGLI1#1) or control shRNA (shCtrl) (n = 3). (**B**) Representative Western blot analysis of GLI1, total- and phospho-STAT3 (tSTAT3 and pSTAT3) and IDO1 expression in WM35 melanoma cells treated as described in (**A**). (**B’)** Relative quantification of IDO1 Western blot bands of four independent experiments. (**C**) qPCR analysis of IDO1 mRNA levels in WM35 melanoma cells treated with or without IFNγ (for 18 h with [10 ng/mL]), and lentivirally transduced with shGLI1 (#1 or #2) or control shRNA (shCtrl) (*n* = 3). (**D**) Western blot analysis of GLI1, total- and phospho-STAT1 (tSTAT1 and pSTAT1) and IDO1 expression in WM35 melanoma cells treated as described in (**C**). (**D’**) Relative quantification of IDO1 Western blot bands of three independent experiments described in (**D**). (**B**, **D**) Of note, WM35 melanoma cells displayed full-length GLI1 at 160 kDa and a splice variant at around 130 kDa on the blot. Active STAT3 and STAT1 signaling were assessed by measuring pSTAT3 and pSTAT1, respectively. Total ERK1/2 (tERK1/2) protein expression was used as loading control. (A, C, D’) One-way ANOVA with Tukey’s multiple-comparison test or (**B’**) Student’s t test was used for statistical analysis (*p < 0.05)

To test and validate our findings in skin cancer models with a documented and pathophysiologically relevant role for IDO1 and oncogenic GLI expression, we shifted our focus to human melanoma cells with JAK/STAT signaling-dependent expression of IDO1 [16, 18, 31]. To address the possible role of endogenous, physiological oncogenic GLI activity, we performed RNA-interference (RNAi)-mediated GLI1 inactivation in human BRAFV600E mutated melanoma cells (WM35) and treated the cells with IL6 [75 ng/mL] for 18 h to activate IDO1 expression via STAT3. IDO1 levels were measured by qPCR analysis and Western blotting (Fig. 3A, B, B’). Successful GLI1 knockdown was verified via qPCR analysis (Fig. S4A). In line with our data in human epidermal cells, RNAi-mediated inhibition of GLI1 expression interfered with the induction of IDO1 expression in response to IL6 signaling on both RNA (Fig. 3A) and protein level (Fig. 3B, B’). Of note, pSTAT3/tSTAT3 levels were not changed upon knockdown of GLI1 (Fig. S4B).

Since IFNγ/STAT1 signaling represents a well-known major and potent inducer of IDO1 expression, we also analyzed the requirement of GLI1 in settings where IDO1 expression is induced by the IFNγ/STAT1 axis. Using two different shRNA constructs, we performed RNAi-mediated knockdown of GLI1 in the BRAFV600E mutant human melanoma cell line WM35 treated for 18 h with IFNγ [10 ng/mL]. GLI1 knockdown was validated via qPCR analysis and Western blotting (Fig. S5A, S5B). In line with our observations of IL6-treated cells, genetic inhibition of GLI1 expression strongly reduced IFNγ-mediated transcriptional induction of IDO1 in WM35 melanoma cells as measured by qPCR (Fig. 3C). Of note, IDO1 protein levels strongly decreased by genetic targeting of GLI1 (Fig. 3B, B’, D, D’), supporting a crucial requirement of GLI1 in the activation of IDO1 expression in response to JAK/STAT signaling induced by IL6 or IFNγ. Knockdown of GLI1 did not change pSTAT1/tSTAT1 levels (Fig. S5C). Similar effects of IDO1 reduction on mRNA transcript and on protein level upon GLI1 knockdown as observed in WM35 cells (Fig. 3C-D’, S5A-C) were also found in WM793B BRAFV600E mutant human melanoma cells (Fig. S6A-E). Of note, WM793B cells were resistant to IL6 treatment (data not shown).

### Pharmacological targeting of the HH/GLI pathway reduces the capacity of melanoma cells to produce kynurenine

**Fig. 4:**
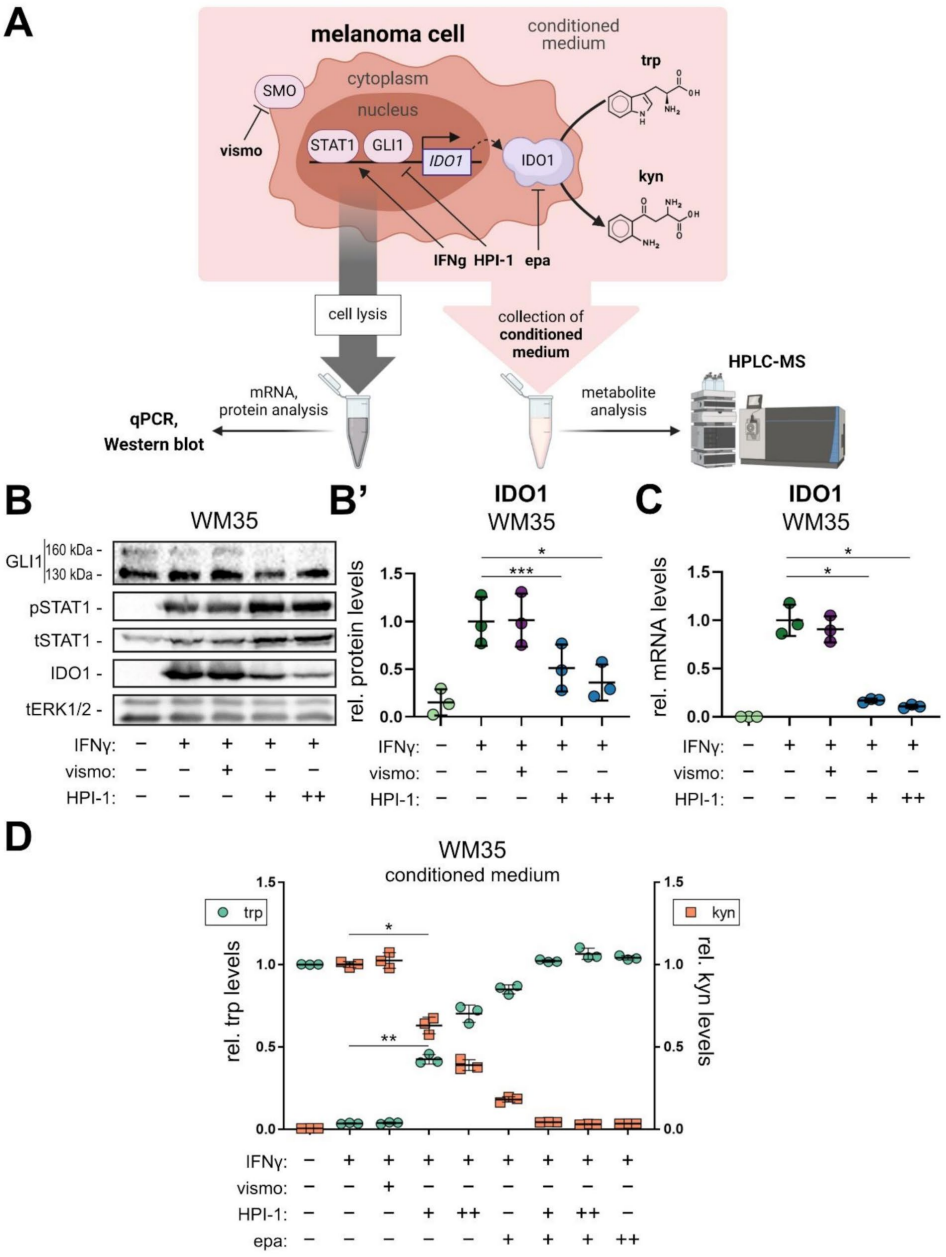
Pharmacological targeting of GLI prevents IFNγ-driven induction of IDO1 and production of immunosuppressive kynurenine metabolites. (**A**) Experimental design of the performed treatments in melanoma cells and downstream analysis. Nuclear STAT1 and GLI1 proteins induce the expression of IDO1, which catabolizes tryptophan to kynurenine, changing their (relative) abundance in the conditioned media of the cells. Melanoma cells were either treated with the HH inhibitors vismodegib or HPI-1 or the IDO1 inhibitor epacadostat. Cells were lysed and analyzed for mRNA and protein expression via qPCR and Western blot and the conditioned medium was collected and analyzed for metabolites via HPLC-MS. (**B**) Representative Western blot analysis of GLI1, total STAT1 (tSTAT1), phospho-STAT1 (pSTAT1) and IDO1 protein levels in WM35 melanoma cells treated with solvent (control), vismo [0.5 µM], HPI-1 [5 µM] (+), [10 µM] (++) for 24 h and/or IFNγ [10 ng/mL] for the last 18 h of the treatment. 130 kDa GLI1 represents a splice variant while the upper (∼ 160 kDa) band marks the transcriptionally active full-length GLI1. Active STAT1 signaling was assessed by measuring pSTAT1. Total ERK1/2 (tERK1/2) protein expression was used as loading control. (**B’)** Relative quantification of IDO1 Western blot bands of three independent experiments described in (**B**). (**C**) qPCR analysis of IDO1 mRNA in WM35 melanoma cells treated as described in (**B**) (*n* = 3, in the control treatment IDO1 was not detectable in two out of three samples). (**D**) HPLC-MS analysis of tryptophan (green) and kynurenine (orange) levels in supernatant of WM35 cells treated as indicated in the graph shown as fold change relative to solvent control or IFNγ, respectively (*n* = 3; vismo [0.5 µM]; HPI-1 [5 µM] (+), [10 µM] (++); epa [0.25 µM] (+), [1.5 µM] (++)). One-way ANOVA with Tukey’s multiple-comparison test was used for statistical analysis (*p < 0.05; **p < 0.01; ***p < 0.001). (vismo: vismodegib; HPI-1: hedgehog pathway inhibitor 1; epa: epacadostat; trp: tryptophan; kyn: kynurenine)

Following the genetic perturbation experiments, we next addressed whether pharmacological targeting of GLI is able to abolish the activation of immunosuppressive IDO1 expression. For this, we treated GLI1-expressing human melanoma cells (WM35) either with the FDA-approved Smoothened-inhibitor vismodegib [0.5 µM] or the GLI inhibitor Hedgehog Pathway Inhibitor-1 (HPI-1) [5 µM, 10 µM] for 6 h and then added IFNγ [10 ng/mL] for another 18 h to induce STAT1 activation and IDO1 expression. mRNA and protein expression of the treated cells were analyzed via qPCR and Western blot (Fig. 4A). While vismodegib treatment did not reduce IDO1 protein or mRNA transcript levels (Fig. 4B-C), treatment with the GLI inhibitor HPI-1 significantly reduced IDO1 expression (Fig. 4B-C). In contrast to HPI-1, vismodegib failed to reduce GLI1 (Fig. S7A), suggesting a crucial role of SMO-independent oncogenic GLI activity in the control of IDO1 expression. Pharmacological inhibition of HH/GLI significantly reduced the ratio of pSTAT1/tSTAT1 levels only with the highest concentration of HPI-1 [10 µM] (Fig. S7B).

Given the critical role of IDO1 in the regulation of tryptophan metabolism, we investigated whether changes in IDO1 levels in response to altered GLI/STAT activity affect extracellular metabolite concentrations. We performed HPLC-MS measurements of the IDO1 substrate tryptophan and its immunosuppressive product kynurenine in conditioned media of human melanoma cells (WM35) treated with either HPI-1, vismodegib or the IDO1-specific inhibitor epacadostat with or without IFNγ (Fig. 4A). Correlating with IDO1 protein levels, kynurenine concentrations were highest in IFNγ plus solvent or IFNγ plus vismodegib treated cells, where tryptophan was quantitatively catabolized resulting in tryptophan depletion (Fig. 4D). By contrast, GLI inhibition by HPI-1 reduced kynurenine and rescued tryptophan levels in a concentration dependent manner. Low concentrations of epacadostat [0.25 µM] partially inhibited kynurenine production, while complete inhibition of kynurenine production was achieved by the combination of low-dose epacadostat [0.25 µM] and HPI-1. Higher concentrations of epacadostat [1.5 µM] also blocked kynurenine production completely, indicating that the observed IFNγ-mediated production of kynurenine was driven by IDO1 (Fig. 4D).

### Rescue of human T cell proliferation upon pharmacological inhibition of the HH/GLI-IDO1 pathway in melanoma cells

**Fig. 5:**
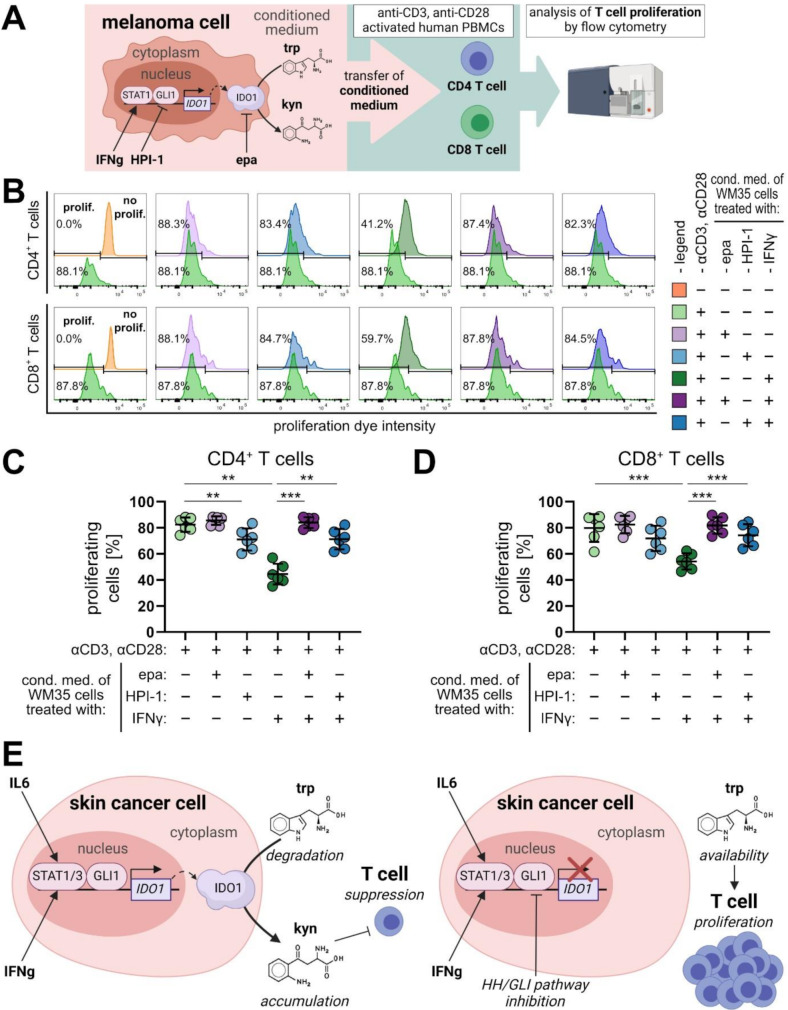
Pharmacological targeting of GLI reverts IDO1/kynurenine-dependent suppression of T cell proliferation. (**A**) Illustration of the experimental design and downstream analysis. Conditioned medium of melanoma cells treated +/- IFNγ [10 ng/mL] and +/- HPI-1 [10 µM] or epacadostat [1.5 µM] for 24 h was transferred on anti-CD3/-CD28 activated human PBMCs in a 1:1 mixture with T cell medium. T cell proliferation was analyzed by flow cytometry after 72 h. (**B**) Representative flow cytometry analysis of anti-CD3/anti-CD28 induced proliferation of human primary CD4^+^ and CD8^+^ T cells of one donor cultured with 50% conditioned medium harvested from treated WM35 melanoma cells as described in (**A**). (**C-D**) Percentage of proliferating CD4^+^ (**C**) and CD8^+^ positive T cells (**D**) of anti-CD3/-CD28 stimulated PBMC cultures from six different human donors after transfer of conditioned medium as described in (**A**) (*n* = 6). (**E**) Graphical summary of the proposed role of GLI-STAT signal integration in cancer immune modulation by IDO1 regulation. Left: Regulation of the *IDO1 cis*-regulatory region in the nucleus of tumor cells. GLI1 in cooperation with STAT1/3 mediates the transcriptional activation of IDO1 in the presence of inflammatory signals such as IL6 or IFNγ. Elevated IDO1 enzyme levels cause intracellular catabolism of tryptophan, which in turn generates high local levels of the immunosuppressive metabolite kynurenine in the tumor microenvironment. High levels of kynurenine efficiently suppress effector T cell activation, thereby interfering with the anti-tumoral immune response. Right: Pharmacological targeting of oncogenic GLI activator forms abrogates the expression of IDO1 despite the presence of IDO1-inducing inflammatory signals such as IL6 or IFNγ. Therefore, GLI targeting results in reduced kynurenine and increased tryptophan concentrations in the tumor microenvironment, which helps reinstating T cell proliferation and anti-tumoral T cell activity. One-way ANOVA with Tukey’s multiple-comparison test was used for statistical analysis (**p < 0.01; ***p < 0.001). (HPI-1: hedgehog pathway inhibitor 1; epa: epacadostat; trp: tryptophan; kyn: kynurenine)

To address the putative immunosuppressive role of the GLI/IDO1 axis on the activation of effector T cells known to play a key role in the anti-tumoral immune response, we investigated whether inhibition of GLI/IDO1 signaling can mitigate the immunosuppressive effects by reducing kynurenine levels. To that end, we generated conditioned media of melanoma cells treated with solvent control, inhibitors of IDO1 or GLI alone or in combination with IFNγ (to induce IDO1 expression and kynurenine production). Conditioned media were then transferred to anti-CD3/anti-CD28 stimulated primary human T cells and their proliferation was analyzed by flow cytometry after 72 h (Fig. 5A). Representative flow cytometry data of CD4^+^ and CD8^+^ T cells treated with the different conditioned melanoma supernatants of one healthy donor are shown in Fig. 5B. The results of a total of six individual donors are depicted in Fig. 5C and D. We found that melanoma cells treated with epacadostat alone did not cause a significant inhibition of CD4^+^ and CD8^+^ T cell proliferation, while HPI-1 alone significantly reduced CD4^+^ (Fig. 5C) but not CD8^+^ T cell proliferation (Fig. 5D). The same effect was observed for the HPI-1 control supernatants. By contrast, conditioned media from IFNγ-treated melanoma cells, which exhibited high levels of kynurenine due to strong IDO1 expression (see Fig. 4B, D), profoundly inhibited the proliferation of CD4^+^ as well as CD8^+^ T cells (Fig. 5C, D). Inhibition of IDO1 by epacadostat in IFNγ-treated melanoma cells completely restored CD4^+^ and CD8^+^ T cell proliferation (Fig. 5C, D), demonstrating that T cell suppression by conditioned medium from IFNγ-treated melanoma cells is caused by increased IDO1 expression and high kynurenine levels (compare Fig. 4B-D). Notably, the activation of CD4^+^ and CD8^+^ T cells was not essentially affected by these treatment conditions, as indicated by equally high levels of CD25^+^ cells (Figure S8A). Treatment of anti-CD3/anti-CD28 stimulated T cells with control medium supplemented with a defined kynurenine concentration led to a comparable inhibition of CD4^+^ and CD8^+^ T cell proliferation serving as further control for kynurenine to be the effector molecule, in line with its documented repressive effect on T cells (Fig. S8B, C) [48–50]. To rule out that remaining amounts of solvent, epacadostat, IFNγ or HPI-1 in the conditioned media account for changes in T cell proliferation independent of their effects on IDO1 activity in melanoma cells, control supernatants containing the respective concentration of epacadostat, IFNγ and HPI-1 alone or in combination were tested on stimulated PBMCs (Fig. S8D). These control experiments clearly show that neither epacadostat [0.75 µM] nor IFNγ [5 ng/mL] affected CD4^+^ or CD8^+^ T cell proliferation, with HPI-1 showing only subtle, yet negative effects alone and in combination with epacadostat and IFNγ (Fig. S8D). Intriguingly and in line with our proposed model of GLI-dependent IDO1 expression, treatment of IFNγ-stimulated melanoma cells with the GLI inhibitor HPI-1 reinstated the proliferation of CD4^+^ and CD8^+^ T cells like epacadostat (Fig. 5C, D), providing functional evidence for a role of HH/GLI signaling as immunosuppressive driver via enhancing IDO1 expression and kynurenine production in the tumor microenvironment (Fig. 5E).

### Induction of IDO1 by cooperative HH/GLI and JAK/STAT signaling in human melanoma and non-melanoma skin cancer patients

To assess the relevance of GLI/STAT-mediated IDO1 expression and immunosuppression to human pathology, we analyzed different available bulk- ([10, 21, 45]) and single cell (for references see Additional file 1 supplementary information) RNA-seq data sets of melanoma and non-melanoma skin cancer patients.

**Fig. 6:**
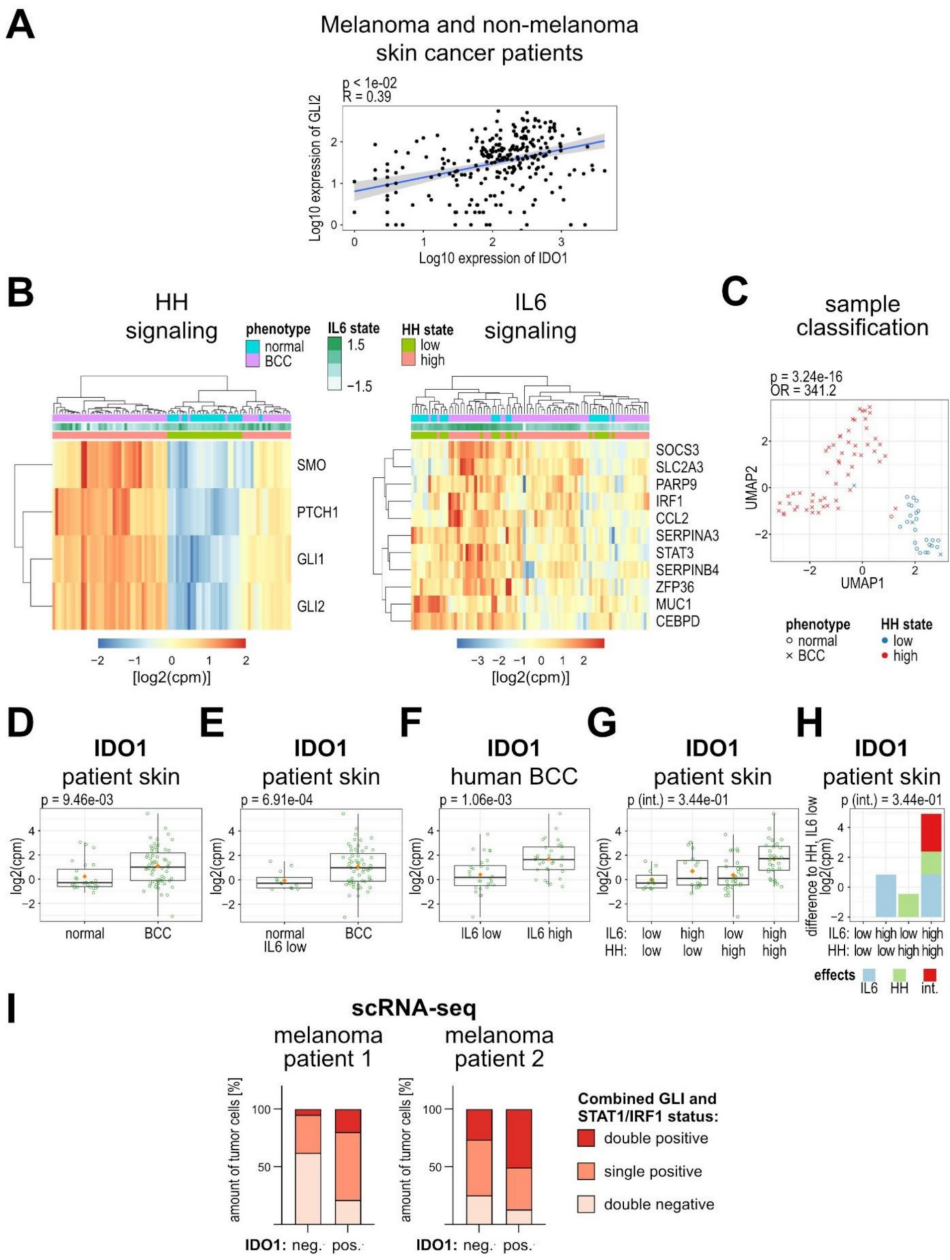
HH-JAK/STAT signaling and IDO1 expression in melanoma and non-melanoma skin cancer patients. (**A**) Spearman correlation between GLI2 and IDO1 expression levels in melanoma and non-melanoma skin cancer patients (*p* < 0.01, *R* = 0.39, *n* = 253). (**B**) Clustering analysis of log2(cpm) mRNA expression values of human BCC and normal skin samples (Bonilla et al., 2016) using HH/GLI (left) and IL6/STAT3 (right) pathway signature genes (*n* = 83). (**C**) Visualization of sample similarity using UMAP, showing HH signaling activity state in normal (circle) and BCC (x) samples (*n* = 83). The *p*-value is obtained from Fisher’s exact test. (**D-G**) Box plots of IDO1 log2(cpm) mRNA expression values grouped into: (**D**) normal (*n* = 24) or BCC (*n* = 59) skin samples; (**E**) normal skin samples with low IL6 signaling activity (*n* = 12) or BCC (*n* = 59) samples; (**F**) BCC samples with low (*n* = 28) or high IL6 signaling activity (*n* = 31); (**G**) low or high HH and IL6 signaling activity (*n* = 83). (**D-F**) *p*-values are obtained from t-tests. (D-G) Orange dots represent mean values. (**H**) Analysis of the HH (green), the IL6 (blue), and the interaction effect (red) of both pathways on IDO1 log2(cpm) mRNA expression values of patient skin samples (*n* = 83) (**G**, **H**) The *p*-value is obtained using a linear model with an interaction coefficient between HH/GLI and IL6/STAT3, encoding the IL6 signature as a binary variable (IL6 high / low). (**I**) Analysis of GLI and STAT1 activity in IDO1^pos^ and IDO1^neg^ primary melanoma cells using single cell RNA sequencing data from two melanoma patients. Tumor cells were grouped as either IDO1^pos^ or IDO1^neg^ based on IDO1 expression and analyzed for the percentage of GLI and/or STAT1/IRF1 expressing cells. Total number of tumor cells: 16,362 (patient 1) and 899 (patient 2). (cpm: counts per million; int.: interaction; OR: odds ratio)

The analysis of a bulk RNA-seq data set with a mixed cohort of melanoma and non-melanoma skin cancer patients revealed a significant positive correlation of IDO1 with GLI2 (*R* = 0.39, *p* < 0.01), supporting an in vivo involvement of the HH/GLI pathway in the induction of IDO1 (Fig. 6A).

To analyze the synergistic up-regulation of IDO1 by the HH/GLI and IL6/STAT3 signaling pathways, we also looked into an RNA-seq data set of human BCC patient as well as normal skin samples (Bonilla et al.) [21]. First, we inferred HH and IL6 pathway activity by defining pathway signatures comprising known pathway target genes (for validation see Additional file 1 supplementary information and Additional file 2 Fig. S9A-F). Next, unsupervised hierarchical clustering was performed to group the sample set according to the HH/GLI and IL6/STAT3 pathway activity status (Fig. 6B). The discrimination between samples with high and low HH/GLI pathway activity exhibited the expected overlap with classified BCC and normal skin samples, respectively, and thus validated our inferred signature (Fig. 6B, S9G). This was confirmed by sample classification using the UMAP dimensionality reduction algorithm (Fig. 6C). As expected, the IL6 low versus high patient cohorts did not discriminate between BCC and normal samples but were equally distributed in both (Fig. 6B, S9H).

As shown in Fig. 6D and E, IDO1 mRNA was higher in human BCC compared to normal skin, which was even more pronounced in comparison to normal skin with low IL6 activity. Also, IDO1 levels were significantly higher in samples with high IL6 pathway activity compared to those with low IL6 activity (Fig. 6F), supporting our findings in vitro. Furthermore, we tested computationally whether IDO1 expression is synergistically induced by HH-IL6 in BCC. We fitted a linear model with an interaction effect between the HH/GLI and IL6/STAT3 effects and observed a clear trend for a synergistic regulation albeit without reaching statistical significance (Fig. 6G, H). We confirmed this trend also in another RNA-seq data set of human BCC patient samples (Fig. S10A-C; GEO accession: GSE58375, Atwood et al.) [10].

Finally, we analyzed scRNA-seq data from two melanoma patients and specifically investigated whether combined HH/GLI and IFNγ/STAT1 signaling would affect the relative amount of IDO1 positive tumor cells. We observed in both patients that IDO1 positive tumor cells displayed a higher percentage of GLI and STAT1/IRF1 double positive cells than IDO1 negative tumor cells. By contrast, IDO1 negative tumor cells mainly had a double negative or single positive status (Fig. 6I). Taken together, these data provide evidence that HH/GLI cooperates with JAK/STAT signaling to synergistically activate the expression of the immunosuppressive enzyme IDO1 in melanoma and non-melanoma skin cancer patients.

## Discussion

We report a novel molecular mechanism by which oncogenic HH/GLI signaling can promote immune evasion through activation of the tryptophan-degrading immunosuppressive enzyme IDO1. Specifically, we found that synergistic interactions of HH/GLI with pro-inflammatory IL6/STAT3 or IFNγ/STAT1 signaling drive high levels of IDO1 expression in melanoma and non-melanoma skin cancers, resulting in suppression of effector T cells via IDO1-mediated production of the immunosuppressive metabolite kynurenine.

Mechanistically, both GLI and STAT transcription factors bind to the *cis*-regulatory region of *IDO1* and cooperatively induce IDO1 transcription that is also accompanied by transcriptionally active histone marks. In addition to its potential therapeutic relevance, the activation of IDO1 in response to HH-IL6 signaling provides a novel mechanism by which pro-inflammatory pathways such as IL6 [51, 52] can elicit a potent immunosuppressive signal in combination with oncogenic HH/GLI. In combination, the tumor-promoting pro-inflammatory activity as well as the immunosuppressive activity may represent crucial molecular processes underlying the potent oncogenicity of combined HH/GLI and IL6/STAT3 signaling.

IFNγ/STAT1 signaling is known to be both a crucial player in anti-tumoral immune responses as well as a strong activator of immunosuppressive IDO1 expression, particularly in melanoma [28, 53, 54]. We therefore investigated whether the HH/GLI signaling pathway also contributes to IFNγ/STAT1 regulated IDO1 expression in melanoma. Like IL6/STAT3, genetic and pharmacological targeting of GLI1 showed that IFNγ/STAT1 requires functional GLI as a second signal for the full-blown induction of IDO1 in human melanoma cells. These findings add to our current understanding of the dual role of IFNγ in the anti-tumoral immune response and in cancer immune evasion and also illustrate how combinatorial signal integration events, such as the cooperation of HH/GLI and JAK/STAT, substantially affect the overall biological outcome. Our in silico RNA-seq data analyses of human normal and malignant skin corroborate the presence of the identified molecular mechanism of a HH/GLI-dependent IDO1 induction in melanoma and non-melanoma skin cancer patients.

Given the preclinical and clinical data of trials of multimodal treatments including IDO1 inhibitors [32, 34–38, 55, 56], our data on GLI/STAT-mediated regulation of IDO1 expression and immunosuppressive activity support the evaluation of novel therapies involving combinations of HH/GLI, JAK/STAT and IDO1 inhibitors for the efficient treatment of malignancies such as advanced or metastatic BCC and melanoma [32]. IDO1 inhibitors have been shown to reduce kynurenine levels and thereby enhance the efficacy of immune checkpoint blockers (ICBs). However, phase III clinical trials failed to show a benefit of IDO inhibitors in combination with ICBs [56]. Whether this setback is due to a lack of predictive biomarkers and patient stratification remains to be determined. Reasons for this failure may range from differences in the patient cohort to incomplete inhibition calling for a better understanding of the complex function and regulation of IDO1 in the tumor and its microenvironment (TME) [55, 57, 58].

The IDO1-kynurenine pathway has been shown to also shape the cellular landscape of the tumor immune microenvironment. For example, IDO inhibits crucial effector functions of cytotoxic T cells, as shown by reduced proliferation, cytokine release as well as degranulation and toxicity of CD8^+^ effector cells upon exposure to high IDO levels [59]. In addition, IDO-dependent tryptophan metabolites were shown to suppress pro-inflammatory Th1 responses [60], indicating that IDO attenuates important effector functions of CD4^+^ and CD8^+^ T cells. In line with these findings, we observe a clear inhibition of T cell proliferation, but not activation, upon culturing human CD4^+^ and CD8^+^ T cells with conditioned media derived from melanoma cells, which show high IDO expression. Kynurenine has also been shown to enhance the formation of immunosuppressive Treg cells via binding to the aryl hydrocarbon receptor [61]. In this context, it is noteworthy that both murine and human BCC, which have been reported to express IDO1 [62], display increased Treg cell numbers [15, 16, 63]. Thus, it will be important to address in future studies the role of GLI/STAT and IDO1 activity in expansion and accumulation of Treg cells in the tumor immune microenvironment. Further, IDO1 activation in melanoma has been demonstrated to recruit myeloid derived suppressor cells (MDSCs) via enhancing Treg numbers, thereby contributing to immune evasion and resistance to immunotherapy (reviewed in [28]). Intriguingly, HH/GLI-induced BCC not only displays increased numbers of Treg cells but has also been shown to be infiltrated by immunosuppressive MDSCs (reviewed in [16, 17]). Together, these data suggest that targeting of HH/GLI and JAK/STAT signaling in combination with IDO1 blockers and ICB may be a promising therapeutic approach for HH-associated malignancies, as such a combination would possibly not only target the oncogenic drivers but also help re-establishing the anti-tumoral immune response with more durable therapeutic effects. To develop and improve such therapeutic approaches it will be key to further elucidate the spatial localization and timepoints of IDO1 induction within tumor compartments or the TME during processes such as anti-tumoral immune responses and tumor progression.

## Conclusions

In conclusion, our findings warrant the future evaluation of rational combination treatments using HH/GLI and JAK/STAT inhibitors in combination with IDO1 blockers and/or ICBs to successfully target malignant growth and restore an efficient anti-tumoral immune response.

## Electronic supplementary material

Below is the link to the electronic supplementary material.

Additional file 1: Contains supplementary information on materials listed as tables and on experimental details in separate sections

Additional file 2: Contains supplementary figures S1-S10

## Data Availability

No datasets were generated or analyzed during the current study.

## Abbreviations

BCC: Basal cell carcinoma
dox: Doxycycline
epa: Epacadostat
ChIP: Chromatin immunoprecipitation
GLI: Glioma-associated oncogene
HaCaT: Human keratinocyte
HH: Hedgehog
HPI-1: Hedgehog Pathway Inhibitor-1
ICB: Immune checkpoint blockers
IDO1: Indoleamine 2,3-dioxygenase 1
IL6: Interleukin 6
IFNγ: Interferon gamma
kyn: Kynurenine
PTCH: Patched
shRNA: Short hairpin RNA
SMO: Smoothened
TME: Tumor microenvironment
Tregs: Regulatory T cells
trp: Tryptophan
