# Supplementary material for: Cooperative Hedgehog/GLI and JAK/STAT signaling drives immunosuppressive tryptophan/kynurenine metabolism via synergistic induction of IDO1 in skin cancer
Source: Cell Commun Signal. 2025 Feb 17;23:91. doi: 10.1186/s12964-025-02101-6 (PMC11834474; doi:10.1186/s12964-025-02101-6)
Supplement: Supplementary file 1 — Additional file 1: Contains supplementary information on materials listed as tables and on experimental details in separate sections [file 12964_2025_2101_MOESM1_ESM.pdf]

Supplementary Information – Material tables

Table S1: Compounds and cytokines

Substance	Manufacturer	Cat. No.
Dimethyl sulfoxide (DMSO)	Merck	D2650
Vismodegib (vismo)	LC Laboratories	V-4050
Hedgehog pathway inhibitor 1 (HPI-1)	Merck	H1542
Epacadostat (epa)	Selleckchem	S7910
Doxycycline (dox)	Merck	D9891
Interleukin 6 (IL6)	Peprotech	200-06
Interferon gamma (IFNγ)	Immunotools	11343534
L-tryptophan (trp)	Merck	T8941
L-kynurenine (kyn)	Merck	K8625

Table S2: Nucleotide sequences of primers

Target	Fw/Rv	Sequence (5'-3')	Company	Application
RPLP0	Fw	GGCACCATTGAAATCCTGAGTGATGTG	Microsynth	RT-qPCR
	Rv	TTGCGGACACCCTCCAGGAAGC		
GLI1 endogenous	Fw	TCTGGACATACCCCACCTCCCTCTG	Microsynth	RT-qPCR
	Rv	ACTGCAGCTCCCCCAATTTTTCTGG		
GLI1	Fw	GCCGTGCTAAAGCTCCAGTGAACACA	Microsynth	RT-qPCR
	Rv	TCCCACTTTGAGAGGCCCATAGCAAG		
IDO1	Fw	TGGGAAGACCCAAAGGAGTTTGCAG	Microsynth	RT-qPCR
	Rv	GACCAGAGCTTTCACACAGGCGTCA		
IDO1 STAT-	Fw	GATCCTTTGCCATTCAGCTACTAA	Microsynth	ChIP-qPCR

bs(i)/GLI-bs(i)	Rv	CCCAGTAATTCCCCTGTTTGA		
IDO1 STAT-bs(ii)	Fw	CGTTGTTCCATGTCTTCACTT	Microsynth	ChIP-qPCR
	Rv	GAGGCTAATACTTTGGGAATC		
IDO1 GLI-bs(ii)	Fw	GGCCAGTATGAGCCTAAGCAG	Microsynth	ChIP-qPCR
	Rv	TTAAGAGAGGCAGTGTGGAATAATG		
IDO1 STAT-bs(i)/GLI-bs(i) region	Fw	GGAATTATTGGGGTTTAGAAAAGGA	Merck	PCR (bisulfite converted DNA)
	Rv	[Btn]-ACAAACTTTAATCATTCTTCCTATAACAT		
	-	GTTGGTTATTTTAAATTAAAGGT	Merck	Pyrosequencing

Fw: forward; Rv: reverse; [Btn]: Biotin tag

Table S3: Antibodies (Western blot, ChIP, Flow cytometry)

Antibody	Dilution	Source	Identifier	RRID	Application
GLI1	1:1000	Cell Signaling Technology	2534	*AB_2294745*	Western blot
STAT3	1:6000	BD Biosciences	610189	*AB_397588*	Western blot
Phospho-STAT3	1:1000	Cell Signaling Technology	9131	*AB_331586*	Western blot
STAT1	1:2000	BD Biosciences	610115	*AB_397521*	Western blot
Phospho-STAT1	1:1000	Cell Signaling Technology	9167	*AB_561284*	Western blot
IDO1	1:1000	Cell Signaling Technology	86630	*AB_2636818*	Western blot
ERK1/2	1:1000	Cell Signaling Technology	9102	*AB_330744*	Western blot
Anti-rabbit, HRP	1:3000	Cell Signaling Technology	7074	*AB_2099233*	Western blot
Anti-mouse, HRP	1:3000	Cell Signaling Technology	7076	*AB_330924*	Western blot

myc-tag	1:100	Cell Signaling Technology	2276	*AB_331783*	ChIP
Normal mouse IgG	same as test ab	Cell Signaling Technology	5415	*AB_10829607*	ChIP
STAT3	5 µg/10 µg chromatin	Santa Cruz Biotechnology	sc-482	*AB_632440*	ChIP
Normal rabbit IgG	same as test ab	Santa Cruz Biotechnology	sc-2027X	*AB_737197*	ChIP
H3K27ac	1:100	Cell Signaling Technology	8173	*AB_10949503*	ChIP
Normal rabbit IgG	same as test ab	Cell Signaling Technology	2729	*AB_1031062*	ChIP
Cell proliferation dye eFluor 450	1:5000	Thermo Fisher Scientific	65-0842-85		Flow cytometry
Fixable viability dye eFluor 780	1:3000	Thermo Fisher Scientific	65-0865-14		Flow cytometry
CD3 (PE)	1:25	Immunotools	21850034	*AB_3073933*	Flow cytometry
CD4 (FITC)	1:50	Thermo Fisher Scientific	11-0048-42	*AB_1633390*	Flow cytometry
CD8 (BV510)	1:50	BD Biosciences	563919	*AB_2722546*	Flow cytometry

Supplementary Information – Experimental Details

Cell culture

Authenticity of all cell lines was verified via short tandem repeat analysis (Leibniz-Institute DMSZ, Braunschweig, Germany). All cell lines were routinely tested for Mycoplasma using PheonixDX Mycoplasma Mix (Procomcure Biotech, Thalgau, Austria).

RNA-seq data and clustering analysis – additional information

Information on data analysis and gene signatures ad Fig. S8

Reads (Fastq files of paired-end RNA-sequencing published by Atwood et al.(1) and obtained from the Gene Expression Omnibus database (GEO accession: GSE58375)) were trimmed for adapter/primer sequences with Trim Galore (https://www.bioinformatics.babraham.ac.uk/projects/trim_galore/), a wrapper tool around FastQC (https://www.bioinformatics.babraham.ac.uk/projects/fastqc/) and cutadapt.(2) Subsequently, reads were aligned to the human reference genome GRCh37/hg19 with annotations from GENCODE (V30lift37) using the STAR software (version 2.7.0e,(3)) to obtain the abundance of reads per gene as counts. Count normalization to the library size and background noise elimination was conducted with the R package EdgeR (version 3.24.3,(4)) resulting in counts per million (cpm). Log2-transformed counts were subjected to data scaling and hierarchical clustering (Ward's minimum variance method) based on signaling pathway gene signatures. Gene signatures for HH/GLI and IL6/STAT3 signaling were established in an unbiased manner based on gene sets from the Molecular Signature Database (MSigDB).(5-7) The HH signature was generated from the genes common to three or more of the following gene sets: PID_HEDGEHOG_GLI_PATHWAY(8), PID_HEDGEHOG_2PATHWAY(8), KEGG_HEDGEHOG_SIGNALING_PATHWAY and HALLMARK_HEDGEHOG_SIGNALING(6) resulting in following genes: *SHH*, *PTCH1*, *SMO*, *GLI1* and *GLI2*. SHH was omitted from the HH signature as its expression levels were too low to be analyzed. The IL6 signature was generated from the genes common to at least two of the following gene sets: DASU_IL6_SIGNALING_UP(9), DAUER_STAT3_TARGETS_UP(10), AZARE_STAT3_TARGETS(11) and a combination of the pathway component gene sets: PID_IL6_7_PATHWAY(8), BIOCARTA_IL6_PATHWAY and ST_STAT3_PATHWAY resulting in following genes: *CCL2*, *CEBPB*, *CEBPD*, *FGG*, *IL6*, *JUN*, *LBP*, *MAFF*, *MCL1*, *MMP3*, *SOCS3*, *SOD2*, *STAT3*, *THBS1* and *ZFP36*. *FGG* and *LBP* were omitted from the IL6 signature as their expression levels were too low to be analyzed.

Information on gene signatures ad Fig. 6B, S7A-H

The validated HH gene signature: *GLI1, GLI2, PTCH1* and *SMO* (generated as described above) was used and further evaluated in this analysis of the Bonilla RNA-seq data set(12). The generated IL6 gene signature: *CCL2*, *CEBPB*, *CEBPD*, *FGG*, *IL6*, *JUN*, *LBP*, *MAFF*, *MCL1*, *MMP3*, *SOCS3*, *SOD2*, *STAT3*, *THBS1* and *ZFP36* (generated as described above) was further evaluated and modified according to the literature and mRNA expression data in IL6-treated human HaCaT keratinocytes. This resulted in following IL6 gene signature:

Table S4: IL6 gene signature.

Gene	Reference
BCL6	(13), *, x
CCL2	(14), **
CEBPD	(15), **
IRF1	(14), *
PARP9	(15), *
MUC1	(14), *
SERPINA3	(16), *
SERPINB4	*
SLC2A3	(14), *
SOCS3	(17), **
STAT3	**
ZFP36	**

* found induced in mRNA expression data
** found induced in mRNA expression data and redundantly occurred in MSigDB gene sets
x … was omitted from the IL6 gene signature since its internal correlation was below the mean correlation (Fig. S7D)

The consistency of both (HH and IL6) gene signatures was validated by calculating correlations of genes within each gene set, which greatly exceeded correlations in randomized control gene sets (Fig. S7A-F).

Single cell RNA-seq data

The Single Cell Portal (https://singlecell.broadinstitute.org/single_cell) was used to obtain single cell data from two separate sample sets.(18, 19)

Promoter analysis

For the prediction of putative GLI binding sites the D-Light client-server software package(20) was fed with the matrix of consensus and non-consensus GLI binding site motives.(21) The ENCyclopedia Of DNA Elements (ENCODE) project was used to derive known STAT binding sites.(22)

Pyrosequencing

Quantitative methylation analysis of the CpG site closest to the STAT binding site (i) and GLI binding site (i) region was performed by bisulfite pyrosequencing as previously described in.(23) Targeted pyrosequencing for quantification of DNA-methylation was performed using the Pyromark Q24 (Qiagen). Assays were designed using the PyroMark Assy Design Software 2.0 (Qiagen) and the PyroMark Q24 Advanced Software 3.0 (Qiagen). Each assay was designed with a built-in bisulfite conversion control to check for successful bisulfite conversion. Methylation values [%] were calculated using the PyroMark Q24 Advanced Software according to quantified RLU values.

RNA interference and lentiviral transduction – additional information

Production of lentiviral particles and transduction was performed according to the protocol described in Kasper et al.(24) with following modifications:

Transfection solution for lentiviral production solution A: 9 µg pMD2G, 9 µg psPAX2, 9 µg respective shRNA construct (Sigma), 1 mL PBS; solution B: 75 µL metafectene pro (Biontex), 1 mL PBS. Solution A was added to solution B resuspended once and incubated for 20 min at room temperature. For production of lentiviral particles 293FT cells were transfected with the mixture. 11 mL of fresh cell culture medium were added on day 3 and day 4.

Modifications to the lentiviral transduction protocol described in Kasper et al.(24): 1.8 mL virus particles were used per 6-well and extended plate centrifugation was conducted for 1.5 h.

For RNA interference lentiviral particles were produced using the following short hairpin RNA (shRNA) constructs purchased from the Mission TRC shRNA Library (Merck, Darmstadt, Germany): control shRNA (SHC002), shGLI1#1 (TRCN0000020486), shGLI1#2 (TRCN0000020488). After transduction cells were selected with puromycin (Merck, Darmstadt, Germany).

HPLC-MS Method for the targeted analysis of kynurenine and tryptophan

Sample preparation

In order to pellet proteins and to extract metabolites, 900 µL ice-cold methanol containing 5.0 µM 3-nitro-L-tyrosine as an internal standard (Sigma-Aldrich, Austria) were added to 100 µL of stored supernatant cell media. Proteins were pelleted by centrifugation at 18620 g and 4.0 °C for 10 minutes. The supernatant was used for HPLC-MS analysis after dilution with ultrapure water at a ratio of 1:5.

Targeted HPLC-MS analysis

An ultra-high performance liquid chromatography (UHPLC) system consisting of an Accela 1250 pump, a Column Oven 300 (all from Thermo Fisher Scientific, Bremen, Germany), and an LC PAL DLW Option Autosampler with a 100 µL syringe (from CTC Analytics AG,

Zwingen, Switzerland) was coupled to a hybrid quadrupole-Orbitrap mass spectrometer (Model Q Exactive™; Thermo Fisher Scientific) equipped with a heated electrospray ion source operating in the positive ion mode. Prior to injection into the HPLC-MS system the injection order was randomized.

In order to monitor potential carry over, a blank run with Millipore water as an injection solute was performed after every third injection of sample. This summed up to 31 sample runs and 12 blank runs.

HPLC settings

For RP-HPLC separations a 100 x 2.1mm inner diameter Hypersil Gold aQ column (Thermo Fisher Scientific) packed with 1.9 µm octadecyl silica particles was applied. For column protection, a 4.0 x 3.0 mm inner-diameter C_{18} Security Guard pre-column (Phenomenex, Torrance, California, USA) was installed.

Mobile phase A and B were Millipore water and acetonitrile, respectively, both containing 0.10 % formic acid (Sigma-Aldrich). The stepped gradient HPLC method started with holding 4.0 % B for 0.50 minutes, followed by the first gradient to 7.0 % B in 2.0 minutes and a second one to 50% B in 1.5 minutes. After washing for 1.0 minutes at 100 % B, the column was re-equilibrated to starting conditions for 3.0 minutes, resulting in a total run time of 8.0 minutes.

A flow rate of 0.30 µL min^{-1} and an injection volume of 2.70 µL were applied. The column temperature was held constant at 30 °C.

MS-settings

A Parallel Reaction Monitoring (PRM) method was applied, with the orbitrap resolution set to 17500, an AGC target of 2e5 and a maximum injection time of 100 ms. The isolation window was set to 1.0 m/z and signals were acquired in profile mode. A scheduled inclusion list with distinctive normalized collision energies (NCEs) was applied for $[M+H^+]$ of kynurenine (209.0920 m/z), tryptophan (205.0970 m/z) and the internal standard 3-nitro-L-tyrosine (227.0660 m/z) as depicted in table 1.

As tune parameters a voltage of 3.2 kV, a capillary temperature of 350°C and an S-lens level of 55 were chosen. Sheath gas and auxiliary gas flow rates were set to 45 and 10 arbitrary units, respectively.

Table S5: Scheduled inclusion list, showing applied time windows and normalized collision energies (NCE) for kynurenine (209.0920 m/z), tryptophan (205.0970 m/z) and nitrotyrosine 8227.0660 m/z)

Mass [m/z]	Polarity	Start [min]	End [min]	NCE
209.0920	Positive	0.00	2.00	35
227.0660	Positive	0.00	2.00	35
205.0970	Positive	0.00	2.00	28
209.0920	Positive	2.00	2.65	35
227.0660	Positive	2.50	3.50	35
205.0970	Positive	3.50	5.00	28
209.0920	Positive	5.00	8.00	35
227.0660	Positive	5.00	8.00	35
205.0970	Positive	5.00	8.00	28

Data evaluation

Acquired .raw files were evaluated via the QuanBrowser in Thermo Xcalibur 3.0.63. The seven most intense fragment ions were used for quantification of tryptophan (118.0653, 132.0815, 144.0803, 146.0604, 159.0908, 170.0593, 188.0712 m/z) and kynurenine (94.0660, 120.0445, 136.0764, 146.0598, 150.0555, 174.0542, 192.0661 m/z), while only four fragment ions were consulted for the quantification of the internal standard 3-nitro-L-

tyrosine (133.0522, 164.0337, 168.0289, 181.0606 m/z). For the quantification of all three analytes, a mass tolerance of 10 ppm, 5 smoothing points and a baseline window of 100 were applied. For tryptophan and kynurenine area noise factor and peak noise factor were set to 1, while for 3-nitro-L-tyrosine values of 2 and 10 were used, respectively. A signal to noise ratio of 9:1 was considered as limit of quantification. Calculated peak areas of tryptophan and kynurenine were normalized to the peak area of 3-nitro-L-tyrosine for each run.

References

1. Atwood SX, Sarin KY, Whitson RJ, Li JR, Kim G, Rezaee M, et al. Smoothened variants explain the majority of drug resistance in basal cell carcinoma. Cancer Cell. 2015;27(3):342-53.

2. Martin M. Cutadapt removes adapter sequences from high-throughput sequencing reads. 2011. 2011;17(1):3.

3. Dobin A, Davis CA, Schlesinger F, Drenkow J, Zaleski C, Jha S, et al. STAR: ultrafast universal RNA-seq aligner. Bioinformatics. 2013;29(1):15-21.

4. Robinson MD, McCarthy DJ, Smyth GK. edgeR: a Bioconductor package for differential expression analysis of digital gene expression data. Bioinformatics. 2010;26(1):139-40.

5. Subramanian A, Tamayo P, Mootha VK, Mukherjee S, Ebert BL, Gillette MA, et al. Gene set enrichment analysis: a knowledge-based approach for interpreting genome-wide expression profiles. Proc Natl Acad Sci U S A. 2005;102(43):15545-50.

6. Liberzon A, Birger C, Thorvaldsdóttir H, Ghandi M, Mesirov JP, Tamayo P. The Molecular Signatures Database (MSigDB) hallmark gene set collection. Cell Syst. 2015;1(6):417-25.

7. Liberzon A, Subramanian A, Pinchback R, Thorvaldsdóttir H, Tamayo P, Mesirov JP. Molecular signatures database (MSigDB) 3.0. Bioinformatics. 2011;27(12):1739-40.

8. Schaefer CF, Anthony K, Krupa S, Buchoff J, Day M, Hannay T, Buetow KH. PID: the Pathway Interaction Database. Nucleic Acids Res. 2009;37(Database issue):D674-9.

9. Dasu MR, Hawkins HK, Barrow RE, Xue H, Herndon DN. Gene expression profiles from hypertrophic scar fibroblasts before and after IL-6 stimulation. J Pathol. 2004;202(4):476-85.

10. Dauer DJ, Ferraro B, Song L, Yu B, Mora L, Buettner R, et al. Stat3 regulates genes common to both wound healing and cancer. Oncogene. 2005;24(21):3397-408.

11. Azare J, Leslie K, Al-Ahmadie H, Gerald W, Weinreb PH, Violette SM, Bromberg J. Constitutively activated Stat3 induces tumorigenesis and enhances cell motility of prostate epithelial cells through integrin beta 6. Mol Cell Biol. 2007;27(12):4444-53.

12. Bonilla X, Parmentier L, King B, Bezrukov F, Kaya G, Zoete V, et al. Genomic analysis identifies new drivers and progression pathways in skin basal cell carcinoma. Nat Genet. 2016;48(4):398-406.

13. Alvarez JV, Frank DA. Genome-wide analysis of STAT target genes: elucidating the mechanism of STAT-mediated oncogenesis. Cancer Biol Ther. 2004;3(11):1045-50.

14. Oh YM, Kim JK, Choi Y, Choi S, Yoo JY. Prediction and experimental validation of novel STAT3 target genes in human cancer cells. PLoS One. 2009;4(9):e6911.

15. Kumari N, Dwarakanath BS, Das A, Bhatt AN. Role of interleukin-6 in cancer progression and therapeutic resistance. Tumour Biol. 2016;37(9):11553-72.

16. Kulesza DW, Ramji K, Maleszewska M, Mieczkowski J, Dabrowski M, Chouaib S, Kaminska B. Search for novel STAT3-dependent genes reveals SERPINA3 as a new STAT3 target that regulates invasion of human melanoma cells. Lab Invest. 2019;99(11):1607-21.

17. Murray PJ. The JAK-STAT signaling pathway: input and output integration. J Immunol. 2007;178(5):2623-9.

18. Wu SZ, Roden DL, Al-Eryani G, Bartonicek N, Harvey K, Cazet AS, et al. Cryopreservation of human cancers conserves tumour heterogeneity for single-cell multi-omics analysis. Genome Med. 2021;13(1):81.

19. Russell AJC, Weir JA, Nadaf NM, Shabet M, Kumar V, Kambhampati S, et al. Slide-tags: scalable, single-nucleus barcoding for multi-modal spatial genomics. bioRxiv. 2023:2023.04.01.535228. Preprint at https://www.biorxiv.org/content/10.1101/2023.04.01.v1. April 03, 2023.

20. Laimer J, Zuzan CJ, Ehrenberger T, Freudenberger M, Gschwandtner S, Lebherz C, Lackner P. D-Light on promoters: a client-server system for the analysis and visualization of cis-regulatory elements. BMC Bioinformatics [Internet]. 2013 2013/04//; 14:[140 p.].

21. Winklmayr M, Schmid C, Laner-Plamberger S, Kaser A, Aberger F, Eichberger T, Frischauf AM. Non-consensus GLI binding sites in Hedgehog target gene regulation. BMC Mol Biol. 2010;11:2.

22. The ENCODE Project Consortium. An integrated encyclopedia of DNA elements in the human genome. Nature. 2012;489(7414):57-74.

23. Sternberg C, Gruber W, Eberl M, Tesanovic S, Stadler M, Elmer DP, et al. Synergistic cross-talk of hedgehog and interleukin-6 signaling drives growth of basal cell carcinoma. International Journal of Cancer. 2018;143:2943 - 54.

24. Kasper M, Regl G, Eichberger T, Frischauf A-M, Aberger F. Efficient Manipulation of Hedgehog/GLI Signaling Using Retroviral Expression Systems. In: Horabin JI, editor. Hedgehog Signaling Protocols. Totowa, NJ: Humana Press; 2007. p. 67-78.
