# Supplementary material for: Cooperative Hedgehog/GLI and JAK/STAT signaling drives immunosuppressive tryptophan/kynurenine metabolism via synergistic induction of IDO1 in skin cancer
Source: Cell Commun Signal. 2025 Feb 17;23:91. doi: 10.1186/s12964-025-02101-6 (PMC11834474; doi:10.1186/s12964-025-02101-6)
Supplement: Supplementary file 2 — Additional file 2: Contains supplementary figures S1-S10 [file 12964_2025_2101_MOESM2_ESM.pdf]

Figure S1

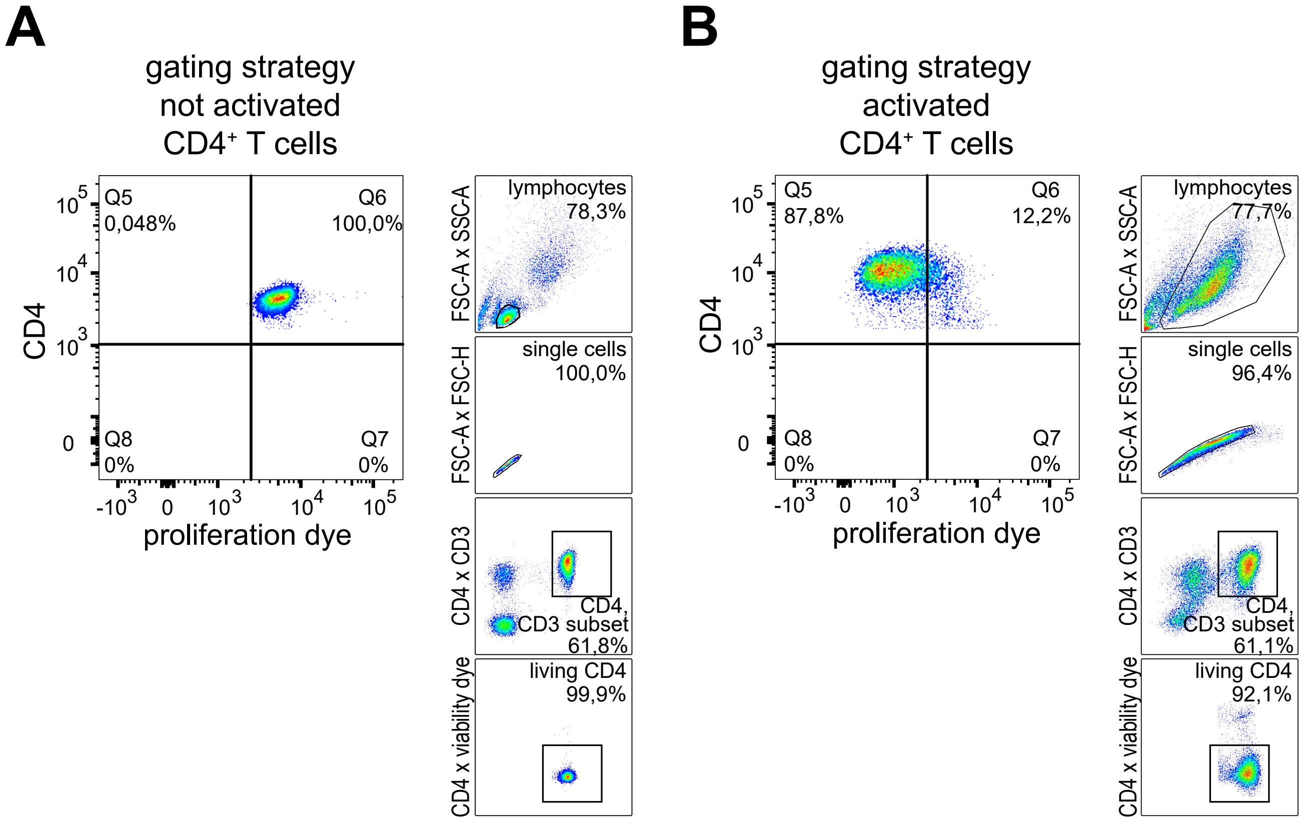

Figure S1: Flow cytometry gating strategy. (A) Gating for the proliferation of $CD4^+$ T cells in unstimulated PBMCs and **(B)** in αCD3, αCD28 activated PBMCs. (A, B) First the lymphocyte population was gated (FSC-A x SSC-A). Then doublet exclusion was performed (FSC-A x FSC-H). Next CD3 and CD4 double positive T cells were gated (CD4 x CD3). After that the living cells were gated from the CD3, CD4 double positive T cells (CD4 x viability dye). Final evaluation of the proliferation of the living CD3, CD4 double positive T cells (proliferation dye x CD4). $CD8^+$ T cells were gated accordingly.

Figure S2

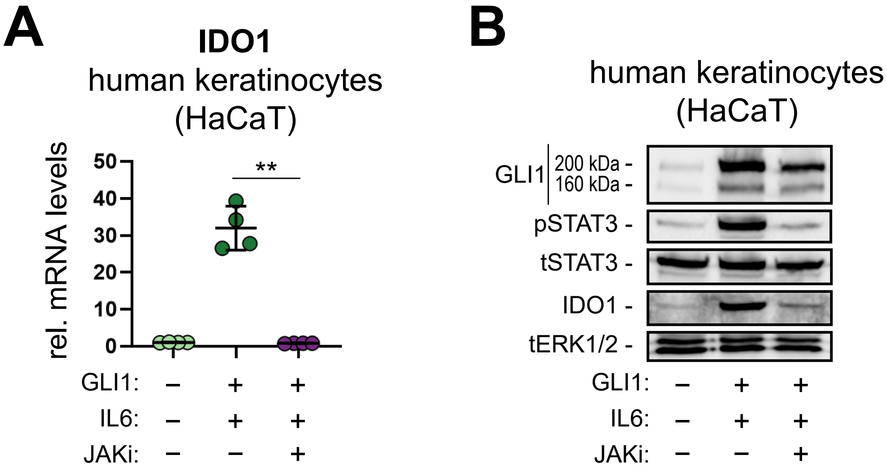

Figure S2: Pan JAK inhibtion reverts the synergistic induction of IDO1 through combined IL6/STAT3 and GLI1 signaling. (A) qPCR analysis of relative IDO1 mRNA levels in dox-inducible GLI1 human HaCaT keratinocytes treated with dox [50 ng/mL] (48 h), JAKi [1 µM] (for the last 24 h) and IL6 [75 ng/mL] (for the last 22 h) as described for Fig. 1 (n = 4). **(B)** Representative Western blot analysis of IDO1 expression in dox-inducible GLI1 human HaCaT keratinocytes treated with dox, JAKi and IL6 as described in (A). Active STAT3 signaling was assessed by measuring phospho-STAT3 (pSTAT3). Total ERK1/2 (tERK1/2) protein expression was used as loading control. Two protein bands for GLI1 are visible representing tagged and untagged GLI1 protein. One-way ANOVA with Tukey's multiple-comparison test was used for statistical analysis (**$p < 0.01$). (dox: doxycycline; JAKi: panJAK inhibitor 1; p: phospho; t: total).

Figure S3

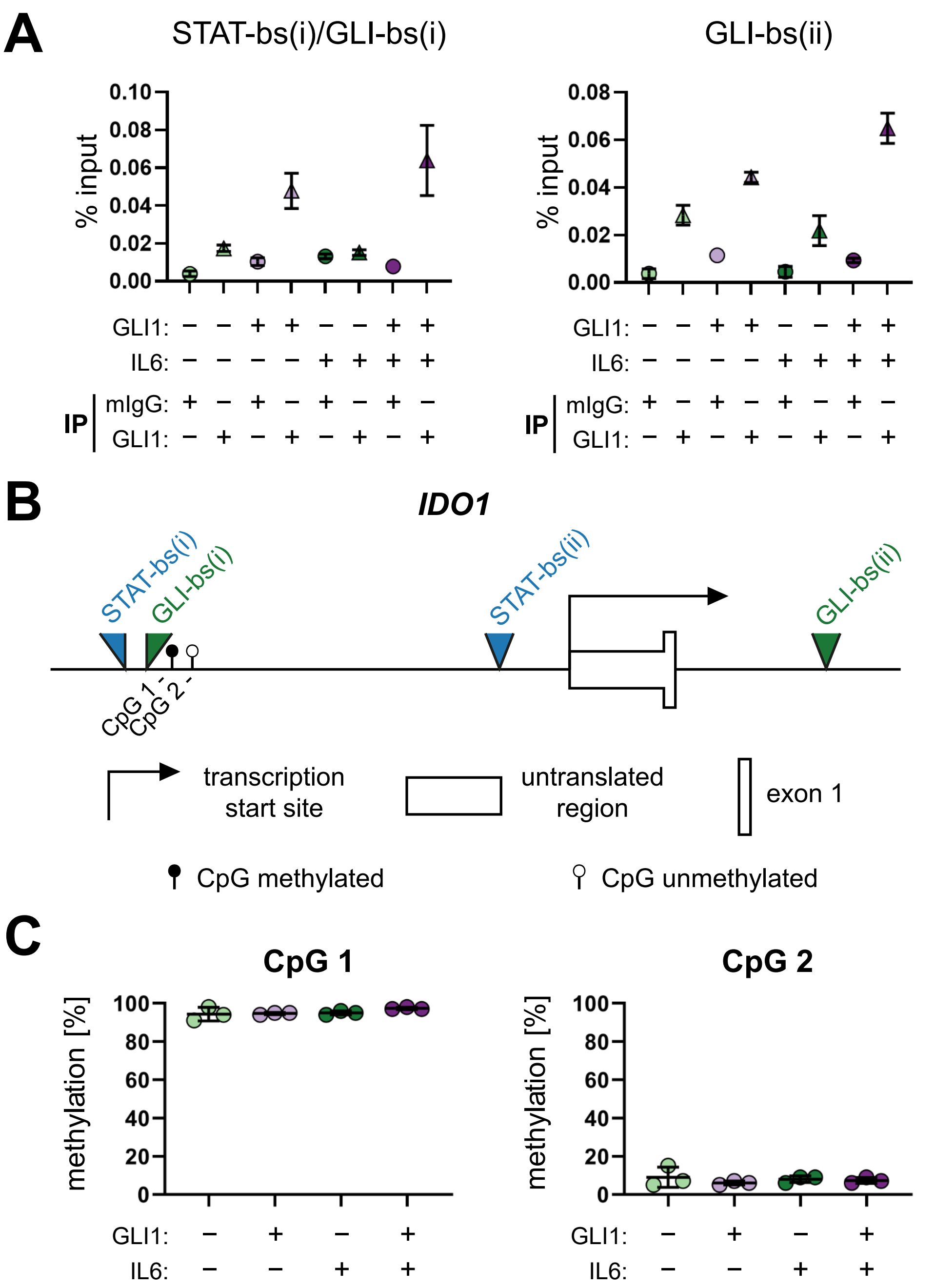

Figure S3: Analysis of GLI1 binding and CpG methylation in the *cis*-regulatory region of IDO1. (A) Targeted ChIP analysis showing GLI1 transcription factor binding to STAT-bs(i)/GLI-bs(i) as well as GLI-bs(ii) in the IDO1 *cis*-regulatory region. Data shown as mean with range. **(B)** Illustration of the *IDO1 cis*-regulatory region with *in silico* predicted binding sites for GLI (green) and STAT (blue) (not drawn to scale). CpG 1 and CpG 2 next to GLI-bs(i) are indicated. Position of CpG 1 is 2815 bp and CpG 2 2781 bp relative to the transcription start site. **(C)** Bisulfite pyrosequencing analysis of the methylation status of CpG 1 and CpG 2 in dox-inducible human keratinocytes (HaCaT). To induce GLI1 expression, cells were pre-treated with dox for 24 h [50 ng/mL] and then treated with IL6 [75 ng/mL] for another 24 h (*n* = 3). One-way ANOVA with Tukey's multiple-comparison test was used for statistical analysis. (dox: doxycycline, mIgG: mouse IgG).

Figure S4

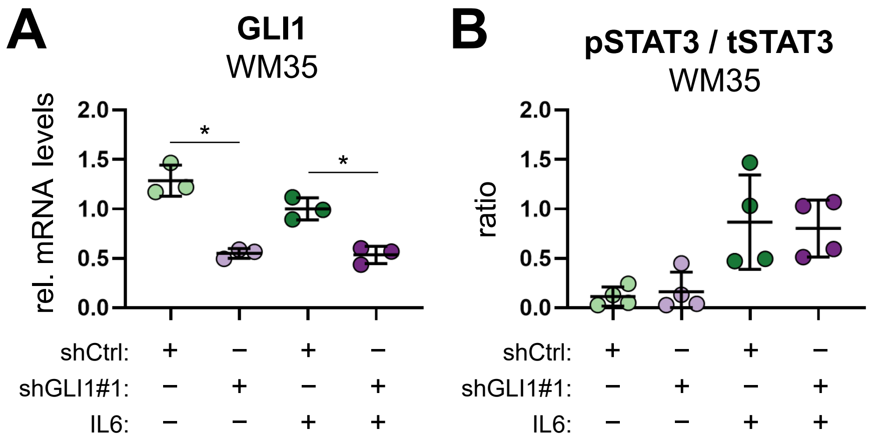

Figure S4: Validated GLI1 RNAi does not affect pSTAT3/tSTAT3 protein levels. (A) qPCR analysis of relative GLI1 mRNA levels (*n* = 3). **(B)** Ratio of pSTAT3 protein levels normalized to tSTAT3 (*n* = 4). Relative quantification of Western blot bands via densitometric image analysis was conducted using Image Lab 4.0 software (Bio-Rad, Vienna, Austria) (*n* = 4). (A, B) WM35 melanoma cells were treated with or without IL6 [75 ng/mL] for 24 h, and lentivirally transduced with shGLI1 (shGLI1#1) or control shRNA (shCtrl). One-way ANOVA with Tukey's multiple-comparison test was used for statistical analysis (*$p < 0.05$). (p: phospho; RNAi: RNA interference; t: total).

Figure S5

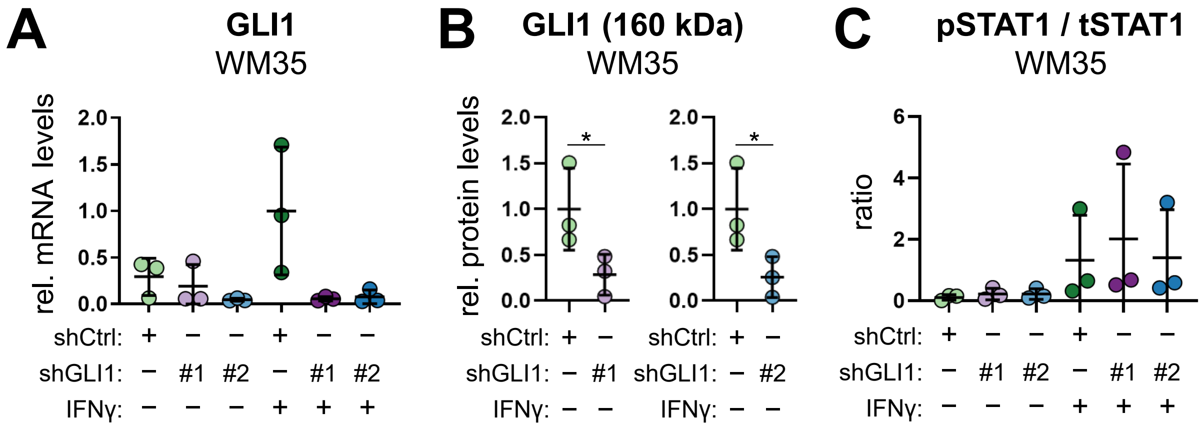

Figure S5: Validated GLI1 RNAi does not affect pSTAT1/tSTAT1 protein levels. (A) qPCR analysis of relative GLI1 mRNA levels in WM35 melanoma cells (n = 3). **(B)** Relative quantification of 160 kDa GLI1 Western blot bands of three independent experiments. Total ERK1/2 (tERK1/2) protein expression served as loading control. **(C)** Ratio of pSTAT1 protein levels normalized to tSTAT1 in WM35 melanoma cells (n = 3). (B, C) Relative quantification of Western blot bands via densitometric image analysis was conducted using Image Lab 4.0 software (Bio-Rad, Vienna, Austria). (A-C) WM35 melanoma cells were treated with or without IFNγ [10 ng/mL] for 18 h, and lentivirally transduced with shGLI1 (#1 or #2) or control shRNA (shCtrl). (A, C) One-way ANOVA with Tukey's multiple-comparison test was used for statistical analysis. (B) Student's t-test was used for statistical analysis (*$p < 0.05$). (p: phospho; RNAi: RNA interference; t: total).

Figure S6

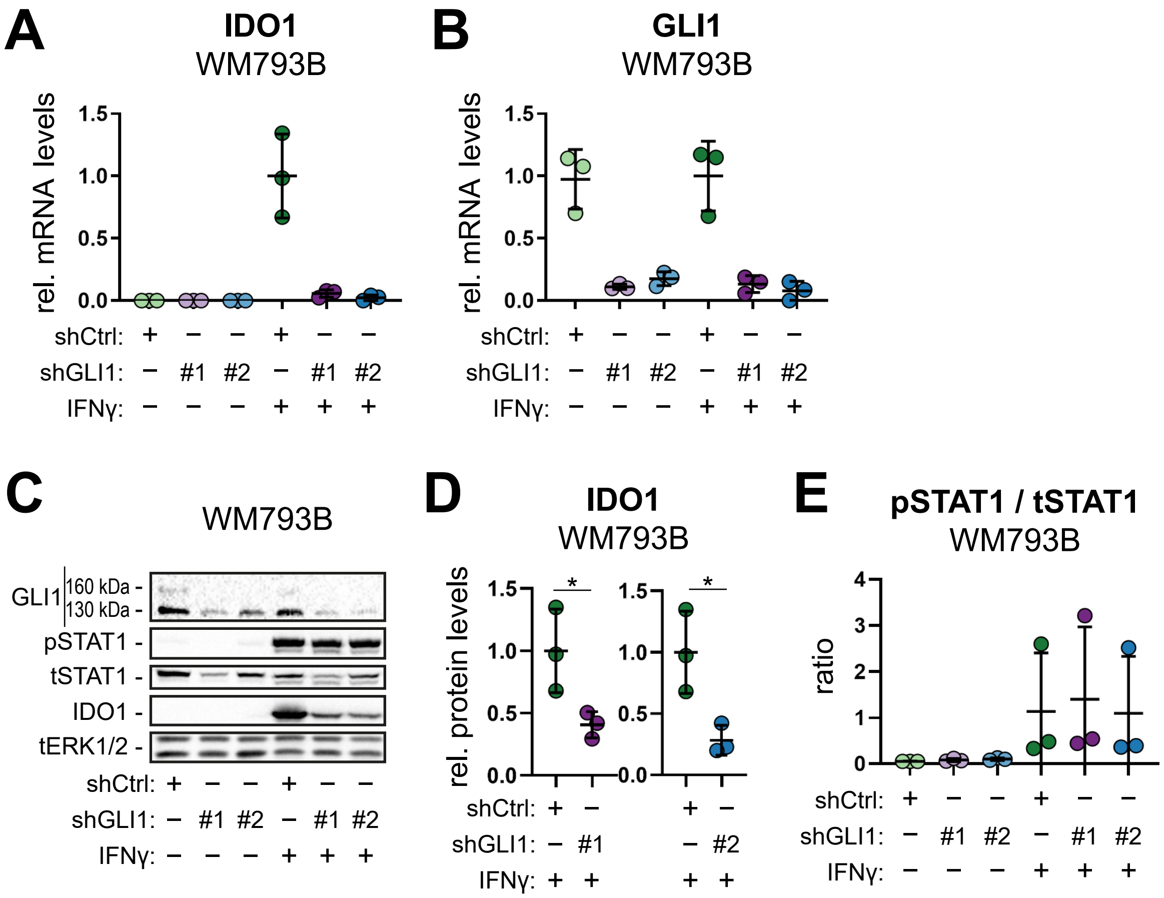

Figure S6: Validated GLI1 RNAi does not affect pSTAT1/tSTAT1 protein levels. (A, B) qPCR analysis of (A) relative IDO1 and (B) relative GLI1 mRNA levels in WM793B melanoma cells (*n* = 3). **(C)** Representative Western blot analysis of GLI1, total- and phospho-STAT1 (tSTAT1 and pSTAT1) and IDO1 expression in WM793B melanoma cells. Of note, WM793B melanoma cells displayed full-length GLI1 at 160 kDa and a splice variant at around 130 kDa on the blot. **(D)** Relative quantification of IDO1 Western blot bands of three independent experiments. Total ERK1/2 (tERK1/2) protein expression served as loading control. **(E)** Ratio of pSTAT1 protein levels normalized to tSTAT1 in WM793B melanoma cells (*n* = 3). Relative quantification of Western blot bands via densitometric image analysis was conducted using Image Lab 4.0 software (Bio-Rad, Vienna, Austria). (A-E) WM793B melanoma cells were treated with or without IFNγ [10 ng/mL] for 18 h, and lentivirally transduced with shGLI1 (#1 or #2) or control shRNA (shCtrl). (A, B, E) One-way ANOVA with Tukey's multiple-comparison test was used for statistical analysis. (D) Student's t-test was used for statistical analysis (*$p < 0.05$). (p: phospho; RNAi: RNA interference; t: total).

Figure S7

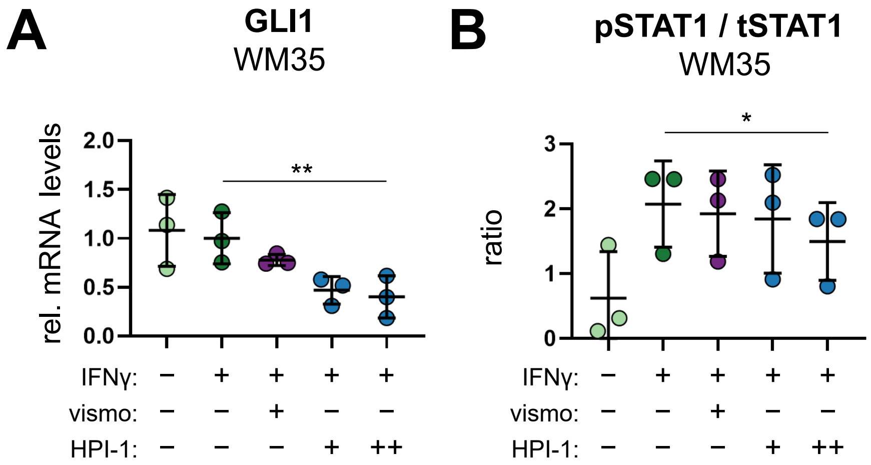

Figure S7: Validation of the effects of the pharmacological inhibitiors vismodegib and HPI-1 on relative GLI1 mRNA levels and pSTAT1/tSTAT1 protein levels. (A) qPCR analysis of relative GLI1 mRNA levels in WM35 melanoma cells (*n* = 3). **(B)** Ratio of pSTAT1 protein levels normalized to tSTAT1 in WM35 melanoma cells (*n* = 3). Relative quantification of Western blot bands via densitometric image analysis was conducted using Image Lab 4.0 software (Bio-Rad, Vienna, Austria). (A, B) WM35 melanoma cells were treated for 24 h with solvent, vismo [0.5 µM], HPI-1 [5 µM] (+), [10 µM] (++) and/or IFNγ [10 ng/mL]. IFNγ was added following inhibitor pretreatment for 6 h. One-way ANOVA with Tukey's multiple-comparison test was used for statistical analysis (*$p < 0.05$; **$p < 0.01$). (HPI-1: hedgehog pathway inhibitor 1; p: phospho; t: total; vismo: vismodegib).

Figure S8

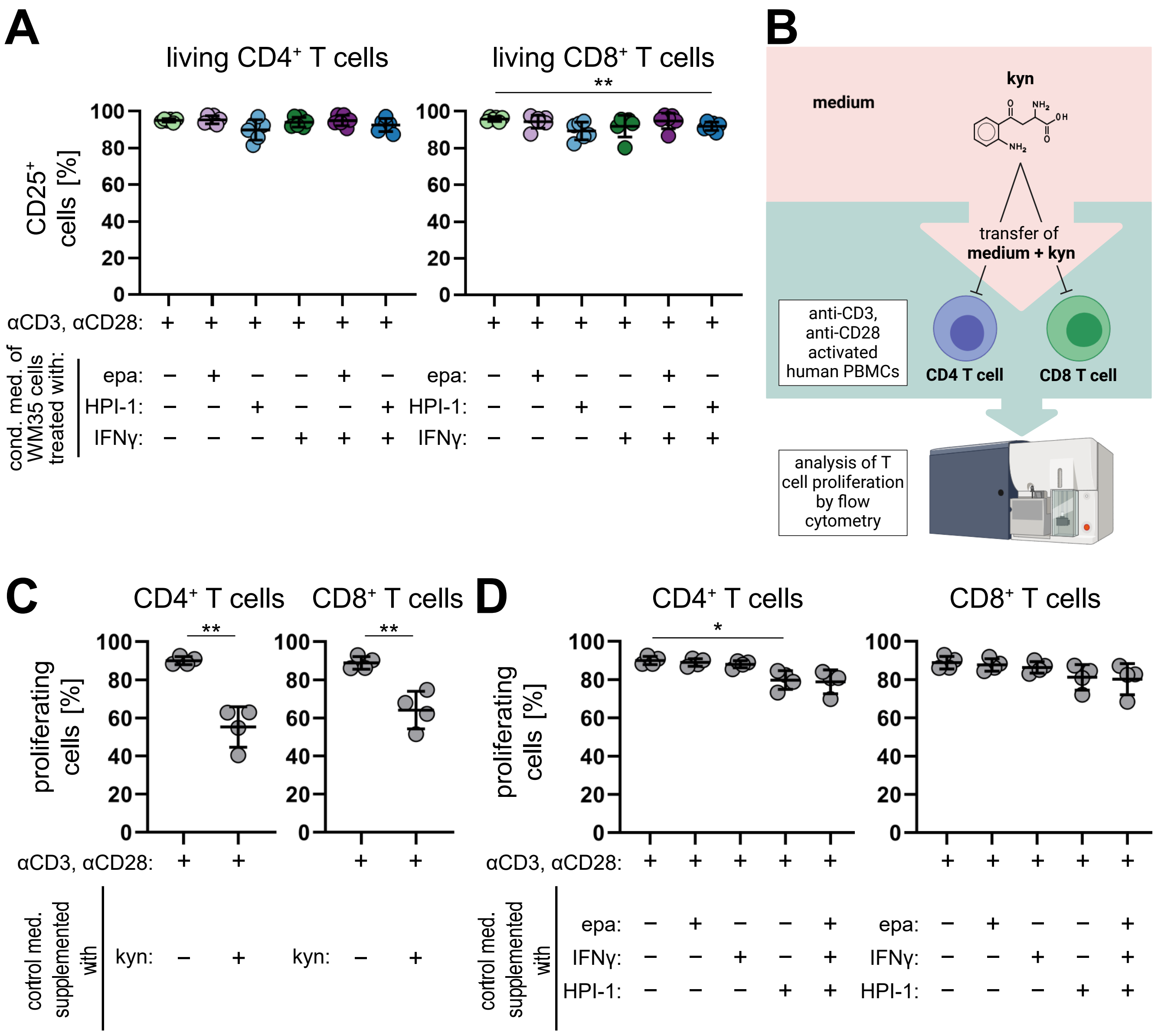

Figure S8: Analysis of CD25 and treatment of PBMCs with control media. (A) Percentage of CD25^{+} cells of living CD4^{+} and CD8^{+} T cells from anti-CD3/-CD28 stimulated PBMC cultures from six different human donors after transfer of conditioned medium as described in (Fig. 5A) (n = 6). **(B)** Illustration of the preparation of kyn supplemented control medium, transfer to αCD3 and αCD28 activated PBMCs and treatment for 72 h with a final treatment concentration of 100 µM kyn, followed by flow cytometric analysis of the proliferation of **(C)** CD4^{+} and CD8^{+} T cells (n = 4). **(D)** PBMCs were also activated with αCD3 and αCD28 and treated for 72 h with control media supplemented with epa, IFNγ or HPI-1, followed by flow cytometric analysis of the proliferation of CD4^{+} and CD8^{+} T cells (n = 4). The final treatment concentrations were: epa [0.75 µM], IFNγ [5 ng/mL], HPI-1 [5 µM]. (B) Student's t-test was used for statistical analysis. (A, C) One-way ANOVA with Tukey's multiple-comparison test was used for statistical analysis (*p < 0.05; **p < 0.01). (epa: epacadostat; HPI-1: hedgehog pathway inhibitor 1; kyn: kynurenine).

Figure S9

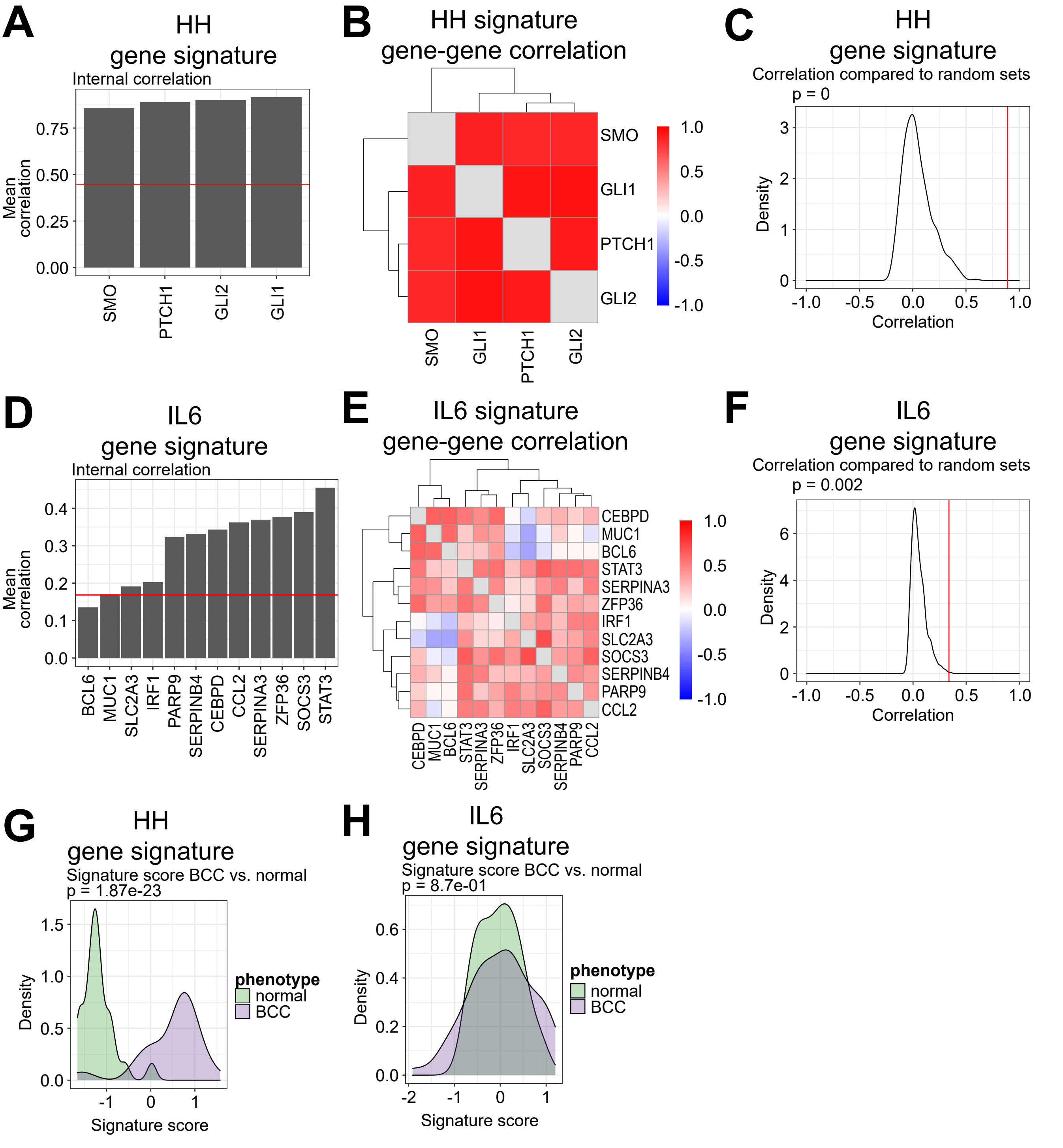

Figure S9: Information on HH and IL6 gene signatures. (A) Internal correlation of the HH signature genes. **(B)** Heatmap of the gene to gene correlation of the HH signature genes. **(C)** Density plot of the correlation of random gene sets with the same number of genes as the HH signature. The red line indicates the correlation of the HH gene signature. **(D)** Internal correlation of the IL6 signature genes. **(E)** Heatmap of the gene to gene correlation of the IL6 signature genes. **(F)** Density plot of the correlation of random gene sets with the same number of genes as the IL6 signature. The red line indicates the correlation of the IL6 gene signature. **(G)** Density plot of the HH signature score in normal (green) and BCC samples (violet). **(H)** Density plot of the IL6 signature score in normal (green) and BCC samples (violet).

Figure S10

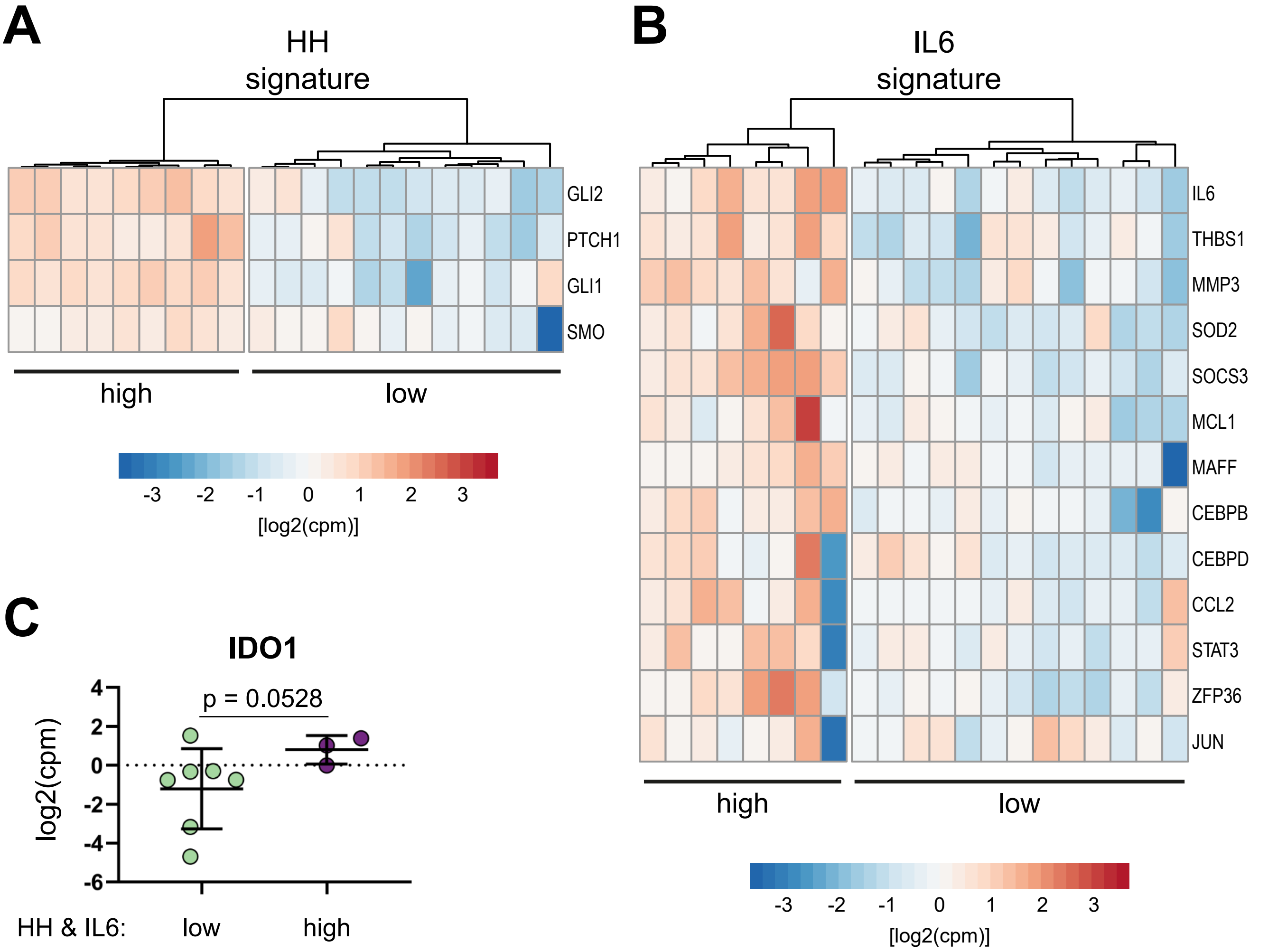

Figure S10: IDO1 is elevated in human BCC patient samples with active HH/GLI and IL6/STAT3 signaling. Clustering analysis of log2(cpm) mRNA expression values of an RNA-seq dataset of human BCC patient and normal skin samples (*n* = 21) from the GEO database (GEO accession: GSE58375) using **(A)** HH/GLI and **(B)** IL6/STAT3 pathway signature genes. **(C)** IDO1 log2(cpm) mRNA expression values of BCC patient and normal skin samples grouped into HH, IL6 low (*n* = 7) or HH, IL6 high (*n* = 3) signaling activity according to the results of the clustering analysis shown in (A) and (B) (*n* = 21). Unpaired Welch's t test was used for statistical analysis (**p* < 0.05). (cpm: counts per million).
